# Changes in Negative Emotions Across Five Weeks of HRV Biofeedback Intervention were Mediated by Changes in Resting Heart Rate Variability

**DOI:** 10.1007/s10484-024-09674-x

**Published:** 2024-11-08

**Authors:** Heidi Jung, Hyun Joo Yoo, Paul Choi, Kaoru Nashiro, Jungwon Min, Christine Cho, Julian F. Thayer, Paul Lehrer, Mara Mather

**Affiliations:** 1 Leonard Davis School of Gerontology, University of Southern California, 3715 McClintock Ave., Los Angeles, CA 90089 USA; 2 University of California, Irvine, Irvine, CA 92697 USA; 3 Rutgers University, Piscataway, 08854 USA; 4 Department of Psychology, University of Southern California, Los Angeles, CA 90089 USA; 5 Department of Biomedical Engineering, University of Southern California, Los Angeles, CA 90089 USA

**Keywords:** Heart rate variability, Emotion, Biofeedback, Slow-paced breathing

## Abstract

Resting heart rate variability (HRV) is typically higher in those with better emotional well-being. In the current study, we examined whether changes in resting HRV mediated changes in negative emotions during a 7-week clinical trial of HRV biofeedback. Younger and older adults were randomly assigned to one of two daily biofeedback practices for 5 weeks: (1) engage in slow-paced breathing to increase the amplitude of oscillations in heart rate at their breathing frequency (Osc+); or (2) engage in self-selected strategies to decrease heart rate oscillations (Osc−). We assessed negative emotion using the State Anxiety Inventory (SAI) and Profile of Mood States (POMS). Resting HRV at pre-intervention was significantly higher among those with lower negative emotion scores. Those participants showing greater increases in resting HRV showed greater decreases in negative emotion. In a mediation model with all participants, resting HRV changes significantly mediated the relationship between training performance (i.e., heart rate oscillation during practice sessions) and changes in negative emotion. However, additional analyses revealed this mediation effect was significantly moderated by condition and was only significant in the Osc+ condition. Thus, resting HRV changes mediated how biofeedback to increase amplitude of heart rate oscillations reduced negative emotion.

**Supplementary Information:**

The online version contains supplementary material available at 10.1007/s10484-024-09674-x.

## Introduction

Heart rate variability (HRV) is a measure of the variation in cardiac beat-to-beat time intervals. In many studies, higher resting HRV is associated with better emotional well-being (Beauchaine & Thayer, 2015; Kemp et al., 2010; Shaffer et al., 2014), whereas lower HRV is associated with poorer emotional and self-rated health (Alvares et al., 2013; Beauchaine & Thayer, 2015; Chalmers et al., 2014; Clamor et al., 2016; Jarczok et al., 2015; Koenig et al., 2016a; Koenig et al., 2016b; Olbrich et al., 2022; Thayer et al., 2012; Thayer et al., 2000; Thayer et al., 2009; Thayer & Lane, 2000, 2009). For example, individuals with higher HRV showed lower levels of worry and rumination, lower anxiety, and generally more regulated emotional responding (Appelhans & Luecken, 2006; Chalmers et al., 2014; Ottaviani et al., 2016). HRV is generally reduced in healthy major depressive disorder (MDD) patients and further diminished in those comorbid with a generalized anxiety disorder (GAD) (Kemp et al., 2012).

The close relationship between HRV and emotions has been explained within the framework of the neurovisceral integration model (Thayer & Lane, 2000). Cardiac vagal control, as assessed by the high-frequency component of HRV (HF-HRV), is believed to reflect the capacity for flexible physiological regulation and is influenced by pathways linking the prefrontal cortex (PFC) with inhibitory medullary cardioacceleratory circuits within a network that includes the anterior cingulate cortex, ventromedial PFC, insular cortex, and amygdala (Thayer & Lane, 2000). The PFC vagal pathways inhibit amygdala activation, suppress sympathoexcitatory neurons in the medulla, and activate vagal motor neurons responsible for parasympathetic activity (Saha, 2005). Consequently, higher HRV indicates better adaptation to external factors, while lower HRV is associated with a higher risk of various disorders, including negative emotions (Alvares et al., 2013; Beauchaine & Thayer, 2015; Chalmers et al., 2014; Clamor et al., 2016; Jarczok et al., 2015; Koenig et al., 2016a; Koenig et al., 2016b; Olbrich et al., 2022; Thayer et al., 2012; Thayer et al., 2000; Thayer et al., 2009; Thayer & Lane, 2000, 2009). A meta-analysis showed low resting HRV is associated with increased amygdala activation and decreased ventromedial PFC activation (Thayer et al., 2012).

Studies examining the relationship between HRV and emotions have generally been cross-sectional and observational, with key variables measured at a single time point without an experimental manipulation of HRV. Among longitudinal studies, some studies measured HRV or emotions at only one time point to investigate their predictive power for other variables measured in the future (Stange et al., 2017; Woody et al., 2014). One study demonstrated that HRV interacts with ruminative thinking to predict future depression (Stange et al., 2017), and another study showed that depression predicts future HRV (Woody et al., 2014). On the other hand, in longitudinal studies that measure HRV and emotions at multiple time points, it is possible to learn how resting HRV changes over time and the relationship between changes in resting HRV and emotional changes, though such studies are rarer. Carnevali et al. (2018) demonstrated the relationship between HRV, rumination, and depressive symptoms over three-timepoints, showing that resting HRV not only predicts future depressive symptoms but also mediates the relationship between rumination and future depressive symptoms. Furthermore, in a longitudinal clinical trial such as the present study, it is possible to compare how change in HRV and emotions relate to each other, thus providing more information about their interrelatedness. Therefore, the primary aim of this study is to investigate the relationship between changes in HRV and changes in negative emotions in this longitudinal clinical trial.

Recent findings suggest that HRV not only reflects the function of brain regions involved in emotion regulation but also influences brain and emotional functions (Mather & Thayer, 2018; Nashiro et al., 2023). Manipulating HRV during daily practice sessions involving slow-paced breathing and HRV biofeedback can improve emotional well-being (Donnelly et al., 2023; Francesca et al., 2021; Goessl et al., 2017; Fernández-Alvarez et al., 2021; Lehrer et al., 2020; Pizzoli et al., 2021). One simple way to increase HRV is to breathe slowly at around a 0.1 Hz rate, which corresponds to approximately six breaths per minute. This breathing pace increases the amplitude of heart rate oscillations at the breathing frequency, potentially due to resonance with blood pressure feedback loops known as the baroreflex (Lehrer & Gevirtz, 2014). Several weeks of daily sessions involving breathing at around 0.1 Hz while getting biofeedback on heart rate and trying to increase the amplitude of heart rate oscillations can decrease depression and anxiety (Goessl et al., 2017; Pizzoli et al., 2021) as well as having other positive psychological effects (Lehrer et al., 2020). One possibility is that the effects of the HRV biofeedback practice may be mediated by the greater parasympathetic activity the practice may stimulate throughout the rest of the day, which in turn improves mood.

Alternatively, the large oscillations in heart rate during HRV biofeedback may strengthen regulatory brain networks involving the medial prefrontal cortex (mPFC) (Mather & Thayer, 2018). Over time, the strengthened brain network dynamic may enhance one’s emotion regulation (Mather & Thayer, 2018). In a recent clinical trial, we showed that 5 weeks of HRV biofeedback training increased connectivity between the left amygdala and medial PFC, as well as overall functional connectivity within emotion-related resting-state networks in younger adults (ClinicalTrials.gov NCT03458910; Heart Rate Variability and Emotion Regulation or “HRV-ER”; Nashiro et al., 2023). Additionally, the increased amygdala-mPFC resting-state functional connectivity mediated the effects of biofeedback on positive emotional memory bias, suggesting that daily practice of enhancing heart rate oscillations can improve implicit emotion regulation by enhancing mPFC coordination of emotion-related circuits (Cho et al., 2023). Therefore, the second aim of this study is to investigate whether the changes in negative emotion across the trial duration are mediated by changes in resting HRV, by changes in functional connectivity in an important emotion-regulation network (amygdala-mPFC coordinated activity), or both.

In the present study, we used data from a recently completed clinical trial of heart rate oscillation biofeedback (ClinicalTrials.gov NCT03458910; HRV-ER). These data are publicly available, and a corresponding data description paper provides additional details (Yoo et al., 2022). This clinical trial primarily focused on the impact of heart rate oscillation biofeedback, involving slow-paced breathing, on emotion-related brain networks (Nashiro et al., 2023). In this study, 106 younger and 59 older individuals underwent a five-week study of daily heart rate oscillation biofeedback sessions. Both intervention groups engaged in two daily practice sessions, lasting between 10 and 20 min. These sessions involved real-time feedback on current heart rates and a 3-min history heart rate display. The two groups had differing objectives: the Increase-Oscillations (Osc+) group aimed to amplify breathing-induced heart rate oscillations by following a slow breathing rhythm guided by a visual pacer. Conversely, the Decrease-Oscillations (Osc−) group was tasked with maintaining a steady heart rate using personal techniques, such as visualizing the ocean, listening to nature sounds, or instrumental music. Participants' negative emotions were assessed using POMS, SAI, and other questionnaires before, during, and after the intervention.

Among younger adults in the HRV-ER trial, the Osc+ condition increased left amygdala-mPFC functional connectivity and functional connectivity in emotion-related resting-state networks during rest after the intervention compared with the Osc− condition (Nashiro et al., 2023). The Osc+ condition also reduced activity in somatosensory brain regions during an emotion down-regulation task, compared with the Osc− condition (Nashiro et al., 2023). The two conditions also had different effects on low-frequency (LF) HRV (Yoo et al., 2022). Specifically, the Osc+ condition increased LF-HRV while the Osc− condition decreased it, an effect mediated by the amplitude of heart rate oscillations achieved during practice sessions (Yoo et al., 2022).

In the HRV-ER clinical trial, there were no significant condition differences in change in self-rated emotions (Nashiro et al., 2023), despite previous findings that this type of biofeedback can decrease anxiety and depression (Donnelly et al., 2023; Lehrer et al., 2020; Pizzoli et al., 2021). One possible reason for the lack of significant differences in emotion changes across conditions could be that participants in the sample were not particularly anxious or depressed at baseline, which may have resulted in a floor effect. Another possibility is that improvements in daily emotional states in those previous HRV biofeedback studies were mediated by intervention-induced increases in vagal HRV during resting states. Indeed, studies that decreased depression generally also increased vagal HRV—measured either as the root mean squared successive differences (RMSSD) or as high-frequency HRV (Donnelly et al., 2023). The lack of significant condition differences in resting-state change in these vagal HRV measures in HRV-ER may explain why we did not see the overall effects of the condition on emotional states.

However, if the beneficial effects of HRV biofeedback on daily emotional states are mediated by changes in resting vagal HRV, we may be able to detect this relationship within the HRV-ER dataset even despite the lack of an overall condition difference. As the relationship between resting HRV and emotion before and after HRV biofeedback training has not yet been examined in this dataset, in this study, we investigated whether changes in vagal HRV and negative emotions are correlated, as well as the potential influence of heart rate oscillation during biofeedback training on pre-post intervention change in negative emotion. To study the association of vagal HRV with emotions, the study focused on RMSSD, as RMSSD has been found to be negatively related to experience of negative emotions (Chalmers et al., 2014; Michels et al., 2013; Ramesh et al., 2023). RMSSD is a time-domain measure of variability between normal heartbeats that mainly reflects parasympathetic activity (rather than sympathetic activity; Elghozi & Julien, 2007). Another reason for using RMSSD as the primary variable for HRV analysis in this study is that RMSSD is less influenced by respiration rate than HF-power (Penttilä et al., 2001). For the performance index of biofeedback training, we extracted the summed power within the 0.063–0.125 Hz range for each participant (corresponding to periods of 8–16 s, a range encompassing paces used by Osc+ participants for their breathing) to obtain a measure of resonance frequency oscillatory activity during biofeedback. This study primarily focused on transient negative emotional states, specifically anxiety assessed with the State Anxiety Inventory (SAI) and mood assessed with the Profile of Mood States (POMS). Additionally, we measured negative emotional traits using the Trait Anxiety Inventory (TAI) and the Center for Epidemiological Studies Depression Scale (CESD).

Research consistently shows that HRV decreases with age, which is associated with reduced autonomic flexibility and a diminished capacity for emotion regulation. Younger adults typically have higher RMSSD, indicating better parasympathetic activity and emotional regulation abilities compared to older adults (Garavaglia et al., 2021; Voss et al., 2015). Additionally, women generally exhibit higher vagally-mediated HRV compared to men, which suggests better parasympathetic activity and greater autonomic flexibility. This difference is observed across various HRV metrics such as RMSSD and HF-HRV (Koenig & Thayer, 2016). The higher HRV in women is often linked to better emotion regulation abilities. Women showed greater vagal activity indexed by HF-power, reflecting greater efficiency in the neural networks involved in autonomic and emotional control (Koenig & Thayer, 2016). Therefore, in addition to the main analysis of this study examining the relationship between HRV and negative emotions and how HRV biofeedback training affects this relationship through changes in resting HRV, we also reported these results separately by age and sex groups.

The structure of this study can be summarized as follows: first, we examined the baseline correlations between HRV and negative emotion scores before the intervention. To examine how changes in HRV from pre- to post-intervention were associated with changes in negative emotion, we performed a partial correlation analysis between resting HRV changes and negative emotion changes from pre to post-intervention timepoints while controlling for age and sex. After examining overall partial correlation results, we compared the results between Osc+ and Osc− conditions. Next, we tested whether resting HRV changes mediated the relationship between HRV biofeedback training performance (as operationalized as resonance frequency power during training relative to during rest) and negative emotion changes, especially in the Osc+ condition. The mediation analysis was conducted separately using SAI and POMS, each also analyzed for all participants together as well as for the Osc+ and Osc− conditions separately. Lastly, we extended the mediation model to include amygdala-mPFC functional connectivity change as a second mediator to compare the mediating roles of resting HRV changes and amygdala-mPFC functional connectivity changes in the relationship between the HRV biofeedback and negative emotion changes.

## Methods

### Participants

We recruited 121 participants in the younger age cohort (18–35 years) and 72 participants in the older age cohort (55–80 years) through diverse recruitment channels, including the USC Healthy Minds community subject pool, an online bulletin board, Facebook, and distribution of flyers. Prior to their participation, individuals provided written informed consent, as approved by the University of Southern California (USC) Institutional Review Board. The participants were organized into small groups, each consisting of 3–6 individuals, meeting consistently at the same time and day weekly. Subsequent to the completion of recruitment and scheduling of group sessions, randomization placed the groups into one of two conditions (see Supplementary Fig. S1 for flow diagram). Following the conclusion of the study, participants received compensation, complemented by bonuses tied to both individual and group performance (uniform incentives for both conditions are elucidated below in the section on "Rewards for Performance"). Screening procedures were implemented for prospective participants, leading to exclusion criteria that encompassed medical, neurological, or psychiatric conditions. Exclusions also applied to individuals with disorders impeding HRV biofeedback procedures (e.g., coronary artery disease, angina, cardiac pacemaker), those presently participating in relaxation, biofeedback, or breathing techniques, and individuals using psychoactive drugs other than antidepressants or anti-anxiety medications. Inclusion criteria allowed for participants utilizing antidepressant or anti-anxiety medication and/or undergoing psychotherapy, provided the treatment had remained constant for a minimum of three months, with no anticipated modifications. Older adults scoring below 16 on the TELE (Gatz et al., 1995) suggesting potential dementia were likewise excluded. After removing all data from excluded people and dropouts, we had 106 younger adults and 59 older adults who completed resting HRV, emotion questionnaires, and training. For the data analysis examining the relationship between resting HRV and negative emotions, data from 100 younger adults and 59 older adults were included (see section "Overview of the statistical analyses" and Supplementary Fig. 1). In the analysis that included resting HRV, negative emotions, and amygdala-mPFC functional connectivity, data from 94 younger adults and 51 older adults were utilized (see section "Overview of the statistical analyses" and Supplementary Fig. 1).

### Procedures

#### Overall Schedule

The study protocol involved seven weekly lab visits and five weeks of home biofeedback training. The first lab visit involved the non-MRI baseline measurements, including various questionnaires. The second lab visit involved the baseline MRI scans and the first session of biofeedback calibration and training. Each of the lab visits started with emotion questionnaires followed by measurement of HRV during a 5-min baseline rest period and progressing to various training conditions to find the best condition. Once calibration was concluded, participants were informed of the most effective strategy and advised to adopt this preferred condition at home for 10 min twice a day for the 1st training week (between the 1st-week visit and the 2nd-week visit), 15 min twice a day for the 2nd training week (between the 2nd week visit and the 3rd week visit), and 20 min twice a day for the remaining weeks (between the 3rd week visit and the 7th week visit). The week-6 lab visit repeated the assessments from the first lab visit. The final (7th) lab visit first repeated the baseline MRI session scans in the same order.

#### Biofeedback Training for the Osc+ Condition

During all practice sessions, participants wore an ear sensor to measure their pulse, observing real-time heart rate biofeedback as they coordinated their inhalation and exhalation with the emWave pacer rhythms. The emWave software (HeartMath®Institute, 2020) provided a summary ‘coherence’ score for participants that was calculated as peak power/(total power—peak power), with peak power determined by finding the highest peak within the range of 0.04–0.26 Hz and calculating the integral of the window 0.015 Hz above and below this highest peak, divided by total power computed for the 0.0033–0.4 Hz range.

During the second lab visit, the calibration to determine their resonance frequency was performed. Individuals were introduced to the device and underwent a series of paced breathing exercises to determine each person's resonance frequency. We identified the resonance frequency for each participant during five minutes of paced breathing at 10, 9, 11, 12, and finally 13 s/breath (Lehrer et al., 2013). After all 5-min breathing segments were complete, we computed various aspects of the oscillatory dynamics for each breathing pace using Kubios HRV Premium 3.1 software (Tarvainen et al., 2014) and estimated which breathing pace best approximated the resonance frequency by assessing which one had the most of the following characteristics: highest low frequency (LF) power, the highest maximum LF amplitude peak on the spectral graph, highest peak-to-trough amplitude, cleanest and highest-amplitude LF peak, highest coherence score and highest RMSSD. Participants were then instructed to train at home with the pacer set to their identified resonance frequency and to try to maximize their coherence scores.

During the third visit, the calibration including 5-min rest was performed. They were asked to complete three 5-min paced breathing segments: the best condition from the last week’s visit, half breath per minute faster and half breath slower than the best condition. They were then instructed to train the following week at the pace that best approximated the resonance frequency based on the characteristics listed above. In subsequent weekly visits, during 5-min training segments, they were asked to try out abdominal breathing and inhaling through nose/exhaling through pursed lips as well as other strategies of their choice.

#### Biofeedback Training for the Osc− Condition

This condition utilized the same ear sensor as the Osc+ condition but paired with custom software designed to provide a contrasting 'calmness' score from the ‘coherence’ score. The calmness score was calculated by multiplying the coherence score that would have been displayed in the Osc+ condition by − 1 adding 10 (an ‘anti-coherence’ score). During each Osc− training session, participants aimed to lower heart rate variability within a specific frequency range, with a calmness score inversely related to their heart rate oscillatory activity. Thus, participants got more positive feedback (higher calmness scores) when their heart rate oscillatory activity in the 0.04–0.26 Hz range was low. More details on the scoring can be found in our data description paper (Yoo et al., 2022).

At the concluding phase of the second lab visit, participants were familiarized with the biofeedback device and asked to devise five different approaches to reduce heart rate variability and oscillations. They were equipped with an ear sensor to monitor heart rate and observed real-time biofeedback as they experimented with each technique for five minutes. Utilizing Kubios for analysis, we determined the most effective strategy based on criteria including the lowest low-frequency (LF) power, minimal LF amplitude peak, reduced peak-to-trough amplitude, and the smallest amplitude of multiple LF peaks, alongside the highest calmness score and lowest RMSSD. Participants were then guided to refine this strategy at home to enhance their calmness scores.

During their third visit, participants were prompted to choose and assess three of their strategies in 5-min intervals. The strategy that aligned closely with the initial calibration criteria was then chosen for continued practice in their home sessions. In further visits, they continued their 5-min practice sessions with the option to explore additional breathing techniques.

#### Weekly Emotion Questionnaire

During each lab visit, participants completed the State Anxiety Inventory (SAI; Spielberger & Gorsuch, 1983) and the Profile of Mood States (POMS; Grove & Prapavessis, 1992) to capture their immediate emotional state. The SAI measures state anxiety using 20 statements. Participants indicated how they felt at the moment on a scale from 1 (not at all) to 4 (very much so). Scores range from 20 to 80, with higher scores correlating with greater state anxiety. We utilized the 40-item version of POMS, where participants rated the extent to which each item currently reflected their feelings on a scale ranging from 1 (not at all) to 5 (extremely). Total mood disturbance was determined by subtracting the total of positive items from negative items, with a constant value (e.g., 100) added to the result to eliminate negative scores. In addition, we administered the Trait Anxiety Inventory (TAI; Spielberger & Gorsuch, 1983) and the Center for Epidemiological Studies Depression Scale (CESD; Radloff, 1977) during weeks 1, 2, 6, and 7 to capture more generalized emotional traits. The TAI measures trait anxiety using 20 statements, which participants rated on a 4-point scale from 1 (not at all) to 4 (very much so). Scores range from 20 to 80, with higher scores correlating with greater trait anxiety. The CESD consists of 20 statements, which participants rated on a 4-point scale from 0 (rarely) to 3 (most or all of the time). Scores range from 0 to 60, with high scores indicating greater depressive symptoms.

#### MRI Scan Parameters

We employed a 3 T Siemens MAGNETOM Trio scanner with a 32-channel head array coil at the USC Dana and David Dornsife Neuroimaging Center. T1-weighted 3D structural MRI brain scans were acquired pre and post intervention using a magnetization prepared rapid acquisition gradient echo (MPRAGE) sequence with TR = 2300 ms, TE = 2.26 ms, slice thickness = 1.0 mm, flip angle = 9°, field of view = 256 mm, and voxel size = 1.0 × 1.0 × 1.0 mm, with 175 volumes collected (4:44 min). Functional MRI scans during resting-state were acquired using multi-echo-planar imaging sequence with TR = 2400 mm, TE 18/35/53 ms, slice thickness = 3.0 mm, flip angle = 75°, field of view = 240 mm, voxel size = 3.0 × 3.0 × 3.0 mm. We acquired 175 volumes (7 min) for the resting-state scans. Participants were instructed to rest, breathe as usual and look at the central white cross on the black screen.

#### Rewards for Performance

Beyond the hourly $15 compensation for each lab session, participants were entitled to additional monetary incentives based on their own and their group's performance. Individually, participants could earn an extra $2 for every time they surpassed their weekly target score, with a maximum limit of 10 instances—a benchmark set by averaging the top ten scores from the previous week's sessions plus 0.3. Group incentives were provided when participants’ group members achieved at least 80% of their prescribed biofeedback training minutes. Specifically, a participant completing their entire training regimen could earn an extra $3 for each group member achieving 100% completion, and $2 for each member reaching at least 80%. These performance-based rewards were computed weekly, with participants informed of their accrued bonuses during each lab visit.

### Data Analysis

#### HRV During Seated Rest

During the pre- and post-intervention lab visits (the second and seventh visits, respectively), HRV was monitored while participants were seated comfortably, knees bent at 90 degrees and feet flat on the ground, for a duration of 5 min. HeartMath emWave pro software, integrated with an infrared pulse plethysmograph (ppg) ear sensor, facilitated the measurement of participants' pulse. Pulse wave was recorded with a sampling rate of 370 Hz, and inter-beat interval data was extracted after eliminating ectopic beats and other artifacts through a built-in process in emWave pro software. We used Kubios HRV Premium Version 3.1 (Tarvainen et al., 2014) to compute three standard heart rate variability metrics: RMSSD, high frequency power (HF-power), and low frequency power (LF-power). RMSSD is the primary resting HRV time domain metric (Laborde et al., 2017; Shaffer & Ginsberg, 2017), as previous research identified it as an indicator of parasympathetic response (Kleiger et al., 2005; Thayer & Lane, 2000). RMSSD is also less affected by respiratory rate than HF HRV (Hill et al., 2009). We also conducted frequency domain analysis using an autoregressive model to derive spectral power in both the HF range (0.15–0.40 Hz) and LF range (0.04–0.15 Hz). Before conducting statistical analyses, the Shapiro–Wilk test confirmed the normal distribution of HRV values. RMSSD, HF power, LF power were not normally distributed (p < 0.05). To correct for this, RMSSD, HF power, and LF power were transformed using the natural log function. We reported basic resting HRV indexes from pre-intervention as baseline measurements. For the analyses examining the relationship between HRV and negative emotion, we used log RMSSD as the main HRV index.

Heart rate data from ear sensors failed to save for the first four participants in the Osc− condition because of technical issues with the first version of the Osc− biofeedback software; therefore, we analyzed HRV data from the remaining 102 younger adults and 59 older adults.

#### Heart Rate Oscillations During Training

We analyzed the training session data from 102 younger adults (5827 sessions) and 59 older adults (4591 sessions). To assess participants’ compliance, we computed the proportion of actual practice time relative to the designated practice time (20 min daily for the initial week, 30 min daily for the second week, and 40 min daily for the third through fifth weeks, culminating in 1190 min total requested practice time). Young adults completed 79% of the stipulated practice time. Specifically, participants in the Osc+ condition achieved 73%, which was significantly lower than those in the Osc− condition, who achieved 85% (*p* = 0.02). Older adults surpassed the requested practice time, reaching 108% overall; within this group, the Osc+ condition attained 112%, while the Osc− condition achieved 104%, with the difference not being statistically significant (*p* = 0.24).

To assess the impact of Osc+ versus Osc− biofeedback during training sessions, we used Kubios HRV Premium 3.1 (Tarvainen et al., 2014) to compute autoregressive spectral power for each training session. We averaged the autoregressive total spectral power from all training sessions for each participant. In addition, we extracted the summed power within the 0.063–0.125 Hz range for each participant (corresponding to periods of 8–16 s, a range encompassing paces used by Osc+ participants for their breathing) to obtain a measure of resonance frequency oscillatory activity during biofeedback.

#### Preprocessing of fMRI Data

To minimize the effects of motion and non-BOLD physiological effects during resting-state fMRI, we employed multi-echo sequences. Research shows that BOLD T2* signal is linearly dependent on echo time, whereas non-BOLD signal is not echo-time dependent (Kundu et al., 2012). Thus, multi-echo acquisitions allow separating of BOLD signal from movement artifact and therefore enhance accuracy of functional connectivity analyses (Dipasquale et al., 2017), with between 2 and 3 times the level of reliability of typical single-echo scans (Lynch et al., 2020). We applied a denoising pipeline using independent components analysis (ICA) and echo-time dependence to distinguish BOLD fluctuations from non-BOLD artifacts including motion and physiology (Kundu et al., 2013).

#### Resting State Functional Connectivity

Seed-based functional connectivity analysis involved defining the mPFC using a previous meta-analysis of brain regions where activity correlated with HRV (Thayer et al., 2012); we used a sphere of 10 mm around the peak voxel, x = 2, y = 46, z = 6. The right and left amygdala were anatomically defined for each participant based on their T1 images. These regions were segmented using FreeSurfer software version 6, which incorporates a longitudinal processing stream to account for the subject-specific correlation of longitudinal data (http://surfer.nmr.mgh.harvard.edu; Fischl et al., 2004). Labels from the specific structures (left/right amygdala) were created as two distinct binary masks in the native space. Each file underwent visual inspection for segmentation accuracy at each time point. We aligned each participant’s preprocessed data to their brain-extracted structural image and the standard MNI 2-mm brain using FSL FLIRT. A low-pass temporal filter of 0–0.1 Hz was applied, and time series were extracted from the mPFC. For each participant, multiple regression analysis was conducted using FSL FEAT, incorporating nine regressors including the mPFC time series, signals from white matter, cerebrospinal fluid, and six motion parameters, resulting in the mPFC connectivity map for each participant.. The amygdalae were then registered to the standard MNI 2-mm brain using FSL FLIRT with trilinear interpolation, followed by thresholding at 0.5 and a binarization process using fslmaths to maintain mask size. From each participant’s mPFC connectivity map, we extracted the mean beta values separately for the right and left amygdalae regions-of-interest (ROIs), which indicate the strength of functional connectivity with the mPFC.

### Overview of the Statistical Analyses

The final common dataset from HRV and emotion data had an N of 100 for younger adults and an N of 59 for older adults (Supplementary Fig. 1). First, to examine the baseline relationship between HRV and emotion before HRV biofeedback training, we ran simple correlation analyses between HRV measures, emotional state scores, SAI and POMS, and emotional trait scores, CESD and TAI at pre-intervention time-point when controlling for age and sex. Then, we examined the baseline relationship separately in each age group and sex group.

To investigate the relationship between changes in resting HRV and changes in negative emotions due to HRV biofeedback training, we performed a correlation analysis between logRMSSD changes and negative emotion changes. Prior study indicated that among HRV indices, RMSSD is less influenced by respiration and is more reliable than HF-HRV (Penttilä et al., 2001). Therefore, we selected logRMSSD as the representative HRV index for subsequent analyses. We used the percent change for all variables. We first calculated the difference in values between both times for each subject before dividing by the values at pre-intervention to normalize the amount of change with respect to pre-intervention. This was then multiplied by 100 to derive a percent change score: ([value_at post_ − value_at pre_]/value_at pre_) × 100. To compare intervention effects in Osc+ and Osc− groups, we separated the conditions and performed correlations. Similarly, we examined the relationship separately in each age group and sex group. To compare the differences in correlation coefficients between the groups, Fisher r-to-z transformations were utilized for significance testing.

Then, we examined whether the relationship between training performance and negative emotions was mediated by change in resting HRV. We measured the training performance using resonance frequency power changes. We calculated resonance frequency power as natural logarithm transformed values of absolute powers of the resonance frequency range (0.063–0.125 Hz: corresponding to periods of 8–16 s) during training. Then, we calculate the change values by calculating the percent change of resonance frequency power compared to resting at pre-intervention to see the mediation effect of resting HRV changes in the relationship between resonance frequency power changes and negative emotion changes. We also examined the moderated mediation effect using age group and sex group as moderators, respectively, to test for age group and sex differences in the mediation model.

Lastly, we further extended our simple mediation model to test for sequential mediation effects. We examined whether the relationship between the independent variable, resonance frequency power during training relative to during rest, and the dependent variable, negative emotional change, was mediated first by the first mediator, resting HRV change, and then by the second mediator, amygdala-mPFC connectivity change. In this mediation model that included amygdala-mPFC connectivity changes, data from 77 younger adults and 68 older adults were used (Supplementary Fig. 1).

We conducted a mediation analysis using the PROCESS macro 4.2 (Hayes, 2017). The simple and sequential mediation models were applied to the SAI and POMS emotion scores separately, each also analyzed for all participants grouped together, as well as separately for Osc+ and Osc− conditions. In each causal model, the unstandardized regression coefficient (c) reflects the total effect. Coefficient c′ reflects the direct effect of the independent variable on the dependent variable absent the mediator. Coefficient a reflects the relationships between the independent variable and the mediator and coefficient b reflects the relationship between the mediator and dependent variable. The product of coefficients (a × b) indicates how much the relationship between the independent variable and the dependent variable is mediated by the mediator (i.e., the indirect effect). Bootstrapping was used for testing mediation hypotheses, using a resampling procedure of 10,000 bootstrap samples (Preacher & Hayes, 2008). Point estimates and confidence intervals (95%) were estimated for the indirect effect. The point estimate was considered significant when the confidence interval did not contain zero.

## Results

### Sample Characteristics

Table 1 provides details about the participants' baseline characteristics. As RMSSD, HF-power, and LF-power were not normally distributed, they were transformed using the natural logarithm.

**Table 1:** Baseline participant characteristics for each condition in each age group at pre-intervention

	Younger (18–35 years)			Older (55–80 years)			Age group difference (t)
	Osc+	Osc−	Condition difference (t)	Osc+	Osc−	Condition difference (t)	
Age (years)	22.80 (2.42)	22.81 (3.25)	− 0.026	64.77 (8.18)	64.93 (5.80)	− 0.083	52.756***
	All: 22.81 (2.80)			All: 64.84 (7.09)			
Sex	29 (M) 27 (F)	22 (M) 22 (F)		9 (M) 22 (F)	8 (M) 20 (F)		
	All: 51 (M) / 49 (F)			All: 17 (M) / 42 (F)			
Mean HR (beat/min)	72.17 (10.35)	72.93 (9.45)	− 0.376	68.84 (8.56)	72.38 (10.81)	− 1.401	− 1.225
	All: 72.51 (9.92)			All: 70.52 (9.77)			
Log RMSSD	4.07 (0.52)	3.96 (0.32)	1.171	3.60 (0.69)	3.42 (0.41)	1.245	− 6.141***
	All: 4.02 (0.44)			All: 3.52 (0.57)			
Log HF-power	6.90 (1.11)	6.75 (0.72)	0.720	5.89 (1.43)	5.40 (1.08)	1.470	− 6.545***
	All: 6.83 (0.96)			All: 5.66 (1.29)			
Log LF-power	7.19 (1.05)	6.88 (0.94)	1.491	6.00 (1.60)	5.14 (1.39)	2.198	− 7.169***
	All: 7.05 (1.01)			All: 5.60 (1.56)			

Means and standard deviations (in parenthesis) are provided. Independent samples t-tests were used to detect condition differences and age group differences

*p < 0.05

**p < 0.01

***p < 0.001, 2-tailed

### Manipulation Check of Training

To examine that the manipulation of condition (Osc+ vs. Osc−) successfully affected the log LF power of heart rate variability during the training sessions, a two-way mixed ANOVA with time-point as a within-subjects factor (2 levels: pre, training) and condition as a between-subjects factor (2 levels: Osc+ , Osc−) was conducted. The interaction between time and condition was significant, *F*(1, 157) = 37.881, *p* < 0.001, *η*_*p*_^2^ = 0.194, indicating that the changes in LF power were differentially influenced by the training conditions. For the Osc+ condition, there was a larger increase in log LF power during training compared to pre-intervention rest, M_pre_ = 6.77, M_training_ = 8.19, *p* < 0.001. For the Osc− condition, there was also a small increase in log LF power during training compared to pre-intervention rest, M_pre_ = 6.21, M_training_ = 6.57, *p* = 0.005, resulting in a significant interaction effect. This result confirms the effectiveness of the manipulation in terms of influencing heart rate oscillations during training, as different conditions led to distinct changes in log LF power.

Next, we examined the effects of the manipulation on resonance frequency power within 0.063–0.125 Hz (corresponding to periods of 8–16 s) during training. The same two-way mixed ANOVA was performed using resonance frequency power and there was a significant interaction between time and condition, *F*(1, 154) = 19.462, *p* < 0.001, *η*_*p*_^2^ = 0.112. Specifically, Osc+ condition showed a larger increase in log resonance frequency power during training compared to pre-intervention baseline, M_pre_ = 5.17, M_training_ = 7.16, *p* < *0.001*, and Osc− condition showed also a small increase, M_pre_ = 4.56, M_training_ = 5.60, *p* = 0.001, resulting in a significant interaction effect.

As expected (as breathing pace fell in the LF range), for log HF power, the interaction between time and group was not statistically significant, *F*(1, 157) = 3.349, *p* = 0.069, *η*_*p*_^2^ = 0.021, suggesting no differential impact of the training conditions on log HF power changes. For logRMSSD, the interaction effect between time and group was not significant, *F*(1, 157) = 0.006, *p* = 0.939, *η*_*p*_^2^ = 0.000, indicating no differential effects between Osc+ and Osc− groups in influencing changes in logRMSSD.

### The Relationship Between HRV and Emotion Scores at Pre-Intervention

We examined the association between HRV at pre-intervention and two emotional trait scores (CESD and TAI) and two emotional state scores (SAI and POMS) at pre-intervention. Higher scores on each of these measures reflects more negative emotion. Table 2 shows baseline correlation coefficients between variables when controlling for age and sex. Log RMSSD and log HF-power showed significant negative correlations with POMS emotion scores. Log LF-power showed negative correlations with SAI and POMS. We also analyzed the correlations between HRV and negative emotion separately for each age group at baseline. Younger adults showed no significant correlations between HRV and emotion scores (Table 3). Older adults showed significant negative correlations between SAI and log RMSSD, log HF-power, and log LF-power, respectively. POMS also was significantly correlated with log RMSSD, log HF-power, and log LF-power (Table 4). Lastly, we analyzed the correlations between HRV and negative emotion separately at baseline for male and female groups. The male group showed no significant correlations between HRV and emotion scores (Table 5). The female group showed significant negative correlations between SAI and log HF-power and between SAI and log LF-power, respectively. POMS also was significantly correlated with log RMSSD, log HF-power, and log LF-power (Table 6).

**Table 2:** Correlation table for resting HRV and SAI, TAI, and CESD at Week 2

Correlations		Mean HR	Log RMSSD at pre	Log HF-power at pre	Log LF-power pre	CESD at pre	TAI at pre	SAI at pre	POMS at pre
Mean HR at pre	*r*	1							
	*p*								
	*df*								
Log RMSSD at pre	*r*	− 0.42^***^	1						
	*p*	< 0.001							
	*df*	155							
Log HF-power at pre	*r*	− 0.47^***^	0.95^***^	1					
	*p*	< 0.001	< 0.001						
	*df*	155	155						
Log LF-power pre	*r*	− 0.41^***^	0.71^***^	0.72^***^	1				
	*p*	< 0.001	< 0.001	< 0.001					
	*df*	155	155	155					
CESD at pre	*r*	0.16*	− 0.08	− 0.03	− 0.11	1			
	*p*	0.044	0.309	0.683	0.185				
	*df*	154	154	154	154				
TAI at pre	*r*	0.11	− 0.11	− 0.08	− 0.13	0.79^***^	1		
	*p*	0.162	0.192	0.304	0.097	< .001			
	*df*	153	153	153	153	152			
SAI at pre	*r*	0.14	− 0.14	− 0.15	− 0.19*	0.63^***^	0.73^***^	1	
	*p*	0.08	0.076	0.063	0.018	< .001	< .001		
	*df*	155	155	155	155	154	153		
POMS at pre	*r*	0.13	− 0.19*	− 0.17*	− 0.18*	0.63***	0.67***	0.83***	1
	*p*	0.105	0.017	0.031	0.025	< .001	< .001	< .001	
	*df*	155	155	155	155	154	153	155	0

Correlations controlled for age and sex

*p < 0.05

**p < 0.01

***p < 0.001, 2-tailed

**Table 3:** Correlation table for resting HRV and SAI, TAI, and CESD at week 2 (pre-intervention) for younger adults

Correlations		Mean HR	Log RMSSD at pre	Log HF-power at pre	Log LF-power pre	CESD at pre	TAI at pre	SAI at pre	POMS at pre
Mean HR at pre	*r*	1							
	*p*								
	*df*	0							
Log RMSSD at pre	*r*	− 0.46	1						
	*p*	< 0.001							
	*df*	96	0						
Log HF-power at pre	*r*	− 0.47	0.95	1					
	*p*	< 0.001	< 0.001						
	*df*	96	96	0					
Log LF-power pre	*r*	− 0.31	0.68	0.65	1				
	*p*	0.002	< 0.001	< 0.001					
	*df*	96	96	96	0				
CESD at pre	*r*	0.19	0.01	0.05	− 0.06	1			
	*p*	0.057	0.939	0.629	0.576				
	*df*	95	95	95	95	0			
TAI at pre	*r*	0.13	− 0.02	− 0.01	− 0.15	0.80	1		
	*p*	0.215	0.875	0.944	0.136	< 0.001			
	*df*	95	95	95	95	94	0		
SAI at pre	*r*	0.02	− 0.02	− 0.04	− 0.08	0.63	0.78	1	
	*p*	0.813	0.843	0.727	0.456	< 0.001	< 0.001		
	*df*	96	96	96	96	95	95	0	
POMS at pre	*r*	0.01	− 0.08	− 0.05	− 0.11	0.63	0.66	0.84	1
	*p*	0.913	0.461	0.6	0.289	< 0.001	< 0.001	< 0.001	
	*df*	96	96	96	96	95	95	96	0

Correlations controlled for age and sex

*p < 0.05

**p < 0.01

***p < 0.001, 2-tailed

**Table 4:** Correlation table for resting HRV and SAI, TAI, and CESD at Week 2 for older adults

Correlations		Mean HR	Log RMSSD at pre	Log HF-power at pre	Log LF-power pre	CESD at pre	TAI at pre	SAI at pre	POMS at pre
Mean HR at pre	*r*	1							
	*p*								
	*df*	0							
Log RMSSD at pre	*r*	− 0.41**	1						
	*p*	0.002							
	*df*	55	0						
Log HF-power at pre	*r*	− 0.53	0.95	1					
	*p*	< 0.001	< 0.001						
	*df*	55	55	0					
Log LF-power pre	*r*	− 0.55	0.77	0.82	1				
	*p*	< 0.001	< 0.001	< 0.001					
	*df*	55	55	55	0				
CESD at pre	*r*	0.10	− 0.2	− 0.14	− 0.18	1			
	*p*	0.452	0.135	0.314	0.184				
	*df*	55	55	55	55	0			
TAI at pre	*r*	0.09	− 0.22	− 0.18	− 0.13	0.77	1		
	*p*	0.494	0.098	0.186	0.325	< .001			
	*df*	54	54	54	54	54	0		
SAI at pre	*r*	0.35**	− 0.3*	− 0.29*	− 0.32*	0.65	0.65	1	
	*p*	0.009	0.023	0.029	0.014	< 0.001	< 0.001		
	*df*	55	55	55	55	55	54	0	
POMS at pre	*r*	0.34*	− 0.33*	− 0.31*	− 0.28*	0.63	0.69	0.82	1
	*p*	0.011	0.011	0.018	0.036	< 0.001	< 0.001	< 0.001	
	*df*	55	55	55	55	55	54	55	0

Correlations controlled for age and sex

*p < 0.05

**p < 0.01

***p < 0.001, 2-tailed

**Table 5:** Correlation table for resting HRV and SAI, TAI, and CESD at week 2 for males

Correlations		Mean HR at pre	Log RMSSD at pre	Log HF-power at pre	Log LF-power at pre	CESD at pre	TAI at pre	SAI at pre	POMS at pre
Mean HR at pre	*r*	1							
	*p*								
	*df*	0							
Log RMSSD at pre	*r*	− 0.44**	1						
	*p*	0.000							
	*df*	63	0						
Log HF-power at pre	*r*	− 0.50**	0.94**	1					
	*p*	0.000	0.000						
	*df*	63	63	0					
Log LF-power at pre	*r*	− 0.45**	0.67**	0.70**	1				
	*p*	0.000	0.000	0.000					
	*df*	63	63	63	0				
CESD at pre	*r*	0.05	− 0.14	− 0.04	− 0.17	1			
	*p*	0.696	0.263	0.745	0.166				
	*df*	63	63	63	63	0			
TAI at pre	*r*	− 0.07	− 0.05	0.02	− 0.13	0.85	1		
	*p*	0.607	0.698	0.883	0.295	0.000			
	*df*	63	63	63	63	63	0		
SAI at pre	*r*	0.01	− 0.08	− 0.07	− 0.09	0.69**	0.81**	1	
	*p*	0.933	0.508	0.602	0.468	0.000	0.000		
	*df*	63	63	63	63	63	63	0	
POMS at pre	*r*	0.01	− 0.13	− 0.07	− 0.11	0.71**	0.75**	0.84**	1
	*p*	0.969	0.322	0.567	0.396	0.000	0.000	0.000	
	*df*	63	63	63	63	63	63	63	0

Correlations controlled for age

*p < 0.05

**p < 0.01

***p < 0.001, 2-tailed

**Table 6:** Correlation table for resting HRV and SAI, TAI, and CESD at week 2 for females

Correlations		Mean HR at pre	Log RMSSD at pre	Log HF-power at pre	Log LF-power at pre	CESD at pre	TAI at pre	SAI at pre	POMS at pre
Mean HR at pre	*r*	1							
	*p*								
	*df*	0							
Log RMSSD at pre	*r*	− 0.41**	1						
	*p*	0.000							
	*df*	87	0						
Log HF-power at pre	*r*	− 0.45**	0.95**	1					
	*p*	0.000	0.000						
	*df*	87	87	0					
Log LF-power at pre	*r*	− 0.38**	0.75**	0.76**	1				
	*p*	0.000	0.000	0.000					
	*df*	87	87	87	0				
CESD at pre	*r*	0.24*	− 0.05	− 0.04	− 0.08	1			
	*p*	0.022	0.616	0.742	0.476				
	*df*	87	87	87	87	0			
TAI at pre	*r*	0.25*	− 0.16	− 0.171	− 0.14	0.76**	1		
	*p*	0.018	0.131	0.109	0.194	0.000			
	*df*	87	87	87	87	87	0		
SAI at pre	*r*	0.26*	− 0.20	− 0.22*	− 0.25*	0.61**	0.68**	1	
	*p*	0.015	0.066	0.038	0.016	0.000	0.000		
	*df*	87	87	87	87	87	87	0	
POMS at pre	*r*	0.23*	− 0.25*	− 0.26*	− 0.23*	0.59**	0.62**	0.79**	1
	*p*	0.031	0.020	0.015	0.029	0.000	0.000	0.000	
	*df*	87	87	87	87	87	87	87	0

Correlations controlled for age

*p < 0.05

**p < 0.01

***p < 0.001, 2-tailed

As an additional analysis, the baseline correlation analysis results for younger males, older males, younger females, and older females are presented in Supplementary Tables 1–4.

### The Relationships Between Changes in HRV and Negative Emotion

**Fig. 1:**
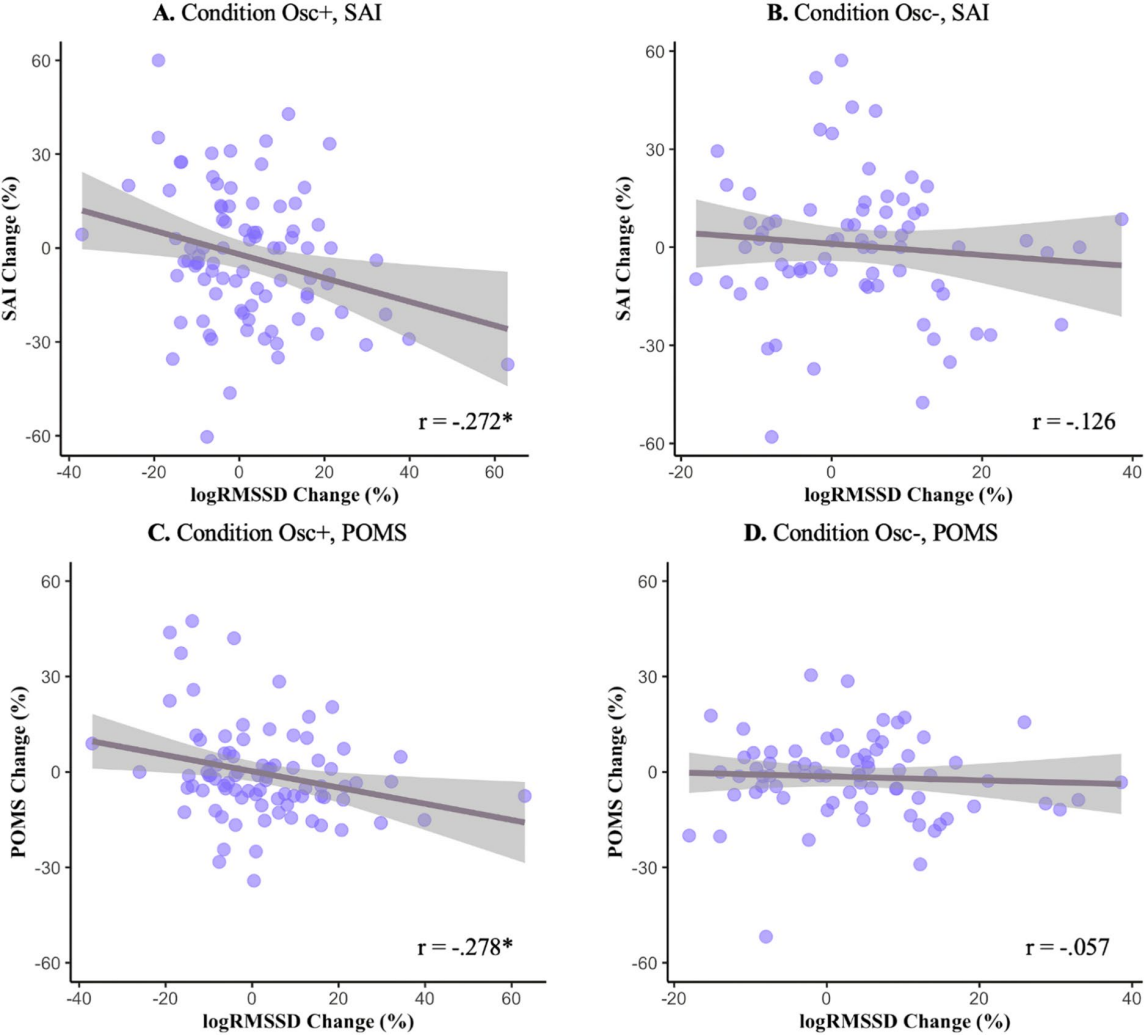
Partial regression plot of week 6 Log RMSSD change and SAI and POMS CHANGES in the Osc+ (AB) and Osc− (CD) conditions

To examine the relationship between change in resting HRV from pre to post and change in negative emotion, we conducted partial correlations between log RMSSD percent change and the SAI and POMS percent change controlling for age (Table 7). Across all participants, there was a significant negative correlation between log RMSSD change and SAI change *r*(156) =  − 0.194, *p* = 0.014. For those in the Osc+ condition, there was a statistically significant negative correlation, *r*(82) =  − 0.266, *p* = 0.013. Those in the Osc− condition did not show a significant correlation, *r*(69) =  − 0.102, *p* = 0.396 (Table 7; Fig. 1). Similarly, across all participants there also was a significant negative correlation between log RMSSD change and the POMS change, *r*(154) =  − 0.188, *p* = 0.019. For those in the Osc+ condition, there was a negative correlation, which was statistically significant, *r*(82) =  − 0.256, *p* = 0.019. Those in the Osc− condition did not show a significant correlation, *r*(69) =  − 0.061, *p* = 0.611 (Fig. 1; Table 7).

**Table 7:** Partial correlation between Log RMSSD change and negative emotion changes from pre to post-intervention

			SAI			POMS			TAI			CESD		
			*r*	*p*	*df*	*r*	*p*	*df*	*r*	*p*	*df*	*r*	*p*	*df*
All	All	All	− 0.194*	0.014	156	− 0.188*	0.019	154	− 0.172*	0.031	155	− 0.089	0.275	152
		Osc+	− 0.266*	0.013	84	− 0.256*	0.019	82	− 0.115	0.293	83	− 0.019	0.867	79
		Osc−	− 0.102	0.396	69	− 0.061	0.611	69	− 0.234	0.052	68	− 0.16	0.182	69
Age group	Younger Adults	All	− 0.197	0.05	97	− 0.294**	0.003	96	− 0.066	0.517	96	− 0.065	0.526	96
		Osc+	− 0.266	0.05	53	− 0.341*	0.012	52	0.051	0.709	53	− 0.004	0.976	52
		Osc−	− 0.13	0.407	41	− 0.2	0.198	41	− 0.222	0.158	40	− 0.142	0.365	41
	Older Adults	All	− 0.199	0.133	56	− 0.035	0.797	55	− 0.304*	0.02	56	− 0.155	0.257	53
		Osc+	− 0.243	0.195	28	− 0.11	0.572	27	− **0.336**	0.069	28	− 0.055	0.787	25
		Osc−	− 0.132	0.513	25	0.07	0.728	25	− 0.309	0.117	25	− 0.302	0.126	25
Sex group	Male	All	0.019	0.876	65	− 0.015	0.908	63	− 0.091	0.467	64	− 0.045	0.722	64
		Osc+	0.033	0.846	35	0.006	0.974	33	0.093	0.584	35	0.186	0.277	34
		Osc−	− 0.029	0.882	27	− 0.095	0.626	27	− 0.307	0.112	26	− 0.246	0.198	27
	Female	All	− 0.323**	0.002	88	− 0.256*	0.015	88	− 0.194	0.067	88	− 0.098	0.365	85
		Osc+	− 0.431**	0.002	46	− 0.367*	0.01	46	− 0.269	0.065	46	− **0.188**	0.216	43
		Osc−	− 0.141	0.378	39	− 0.018	0.909	39	− 0.145	0.366	39	− 0.085	0.596	39

Correlations controlled for age. Values in bold indicate significant age group or sex differences in correlation coefficients

*p < 0.05

**p < 0.01, 2-tailed

When we analyzed the relationship between HRV changes and negative emotion changes separately for younger and older adults across all participants, there was a significant negative correlation between log RMSSD change and POMS change for younger adults, *r*(96) =  − 0.294, *p* = 0.003. For younger adults in the Osc+ condition, there was a statistically significant negative correlation, *r*(52) =  − 0.341, *p* = 0.012. The older adults showed a significant correlation between log RMSSD change and TAI, *r*(56) =  − 0.304, *p* = 0.02 (Table 7). When we compared the correlation coefficients between younger and older adults, the difference of coefficients in TAI between younger and older adults in Osc+ condition was significant, *z* = 1.715, *p* = 0.043.

When we analyzed the relationship between HRV changes and negative emotion changes separately for the male and female groups, there were significant negative correlations only for the female group. There was a significant negative correlation between log RMSSD change and SAI change *r*(88) =  − 0.323, *p* = 0.002. For females in the Osc+ condition, there was a statistically significant negative correlation, *r*(46) =  − 0.431, *p* = 0.002. The females in the Osc− condition did not show a significant correlation, *r*(39) =  − 0.141, *p* = 0.378 (Table 7). Similarly, for females, there also was a significant negative correlation between log RMSSD change and the POMS change, *r*(88) =  − 0.256, *p* = 0.015. For those in the Osc+ condition, there was a negative correlation, which was statistically significant, *r*(46) =  − 0.367, *p* = 0.01. Those in the Osc− condition did not show a significant correlation, *r*(39) =  − 0.018, *p* = 0.909 (Table 7). To examine whether there are differences between the correlation coefficients of male and female groups, Fisher r-to-z transformations were performed. When we compared the correlation coefficients between male and female, the difference of coefficients in SAI between male and female groups was significant, *z* = 2.164, *p* = 0.015. In Osc+ condition, significant sex differences in correlation coefficients were found in SAI (*z* = 2.203, *p* = 0.014), POMS (*z* = 1.743, *p* = 0.041), and CESD (*z* = 1.687, *p* = 0.046).

Additionally, to assess whether changes in HRV during training are correlated with changes in negative emotions, we conducted partial correlations controlling for age. The analyses included the percent change in log RMSSD, log HF power, log LF power, and log resonance frequency power with the percent change in CESD Week 6, SAI Week 6, TAI Week 6, and POMS Week 6 scores. However, no significant correlations were found between changes in log RMSSD, log HF power, log LF power, or log resonance frequency power and changes in CESD Week 6, SAI Week 6, TAI Week 6, or POMS Week 6.

In summary, these results indicate that an increase in resting log RMSSD is associated with a reduction in negative emotions, particularly with lower SAI and POMS scores. In addition, none of the HRV measures during the HRV intervention training showed a significant correlation with negative emotions. Thus, the key factor influencing emotion could be how the intervention training affected resting HRV. In the following section, we test this possibility using mediation models.

### Simple Mediation Model of Resting HRV on Negative Emotions

**Fig. 2:**
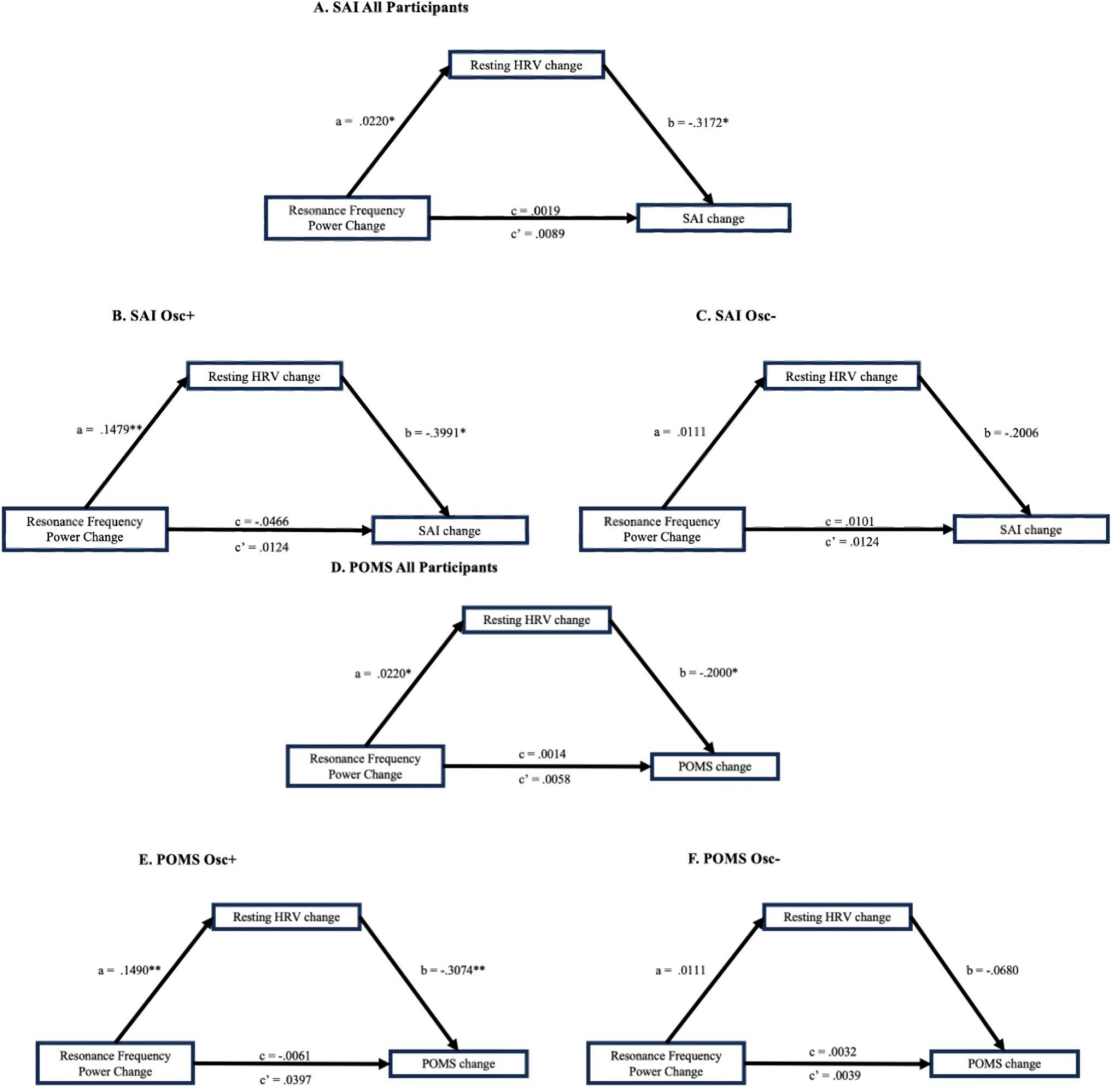
Causal models for SAI change (**A** all participants, **B** Osc+, **C** Osc−) and POMS change (**D** all participants, **E** Osc+, **F** Osc−). Resonance frequency power change: percent change of resonance frequency power during training compared to resonance frequency power at pre-intervention rest; resting HRV change: percent change of log RMSSD at post compare to pre; SAI, percent change of state anxiety at post compared to pre; POMS, percent change of mood disturbance at post compared to pre intervention

To test whether increases in heart rate oscillation during practice directly accounted for decreases in negative mood or whether the effects were the indirect result of changes in resting HRV, we conducted a mediation analysis using bootstrapping method Model 4 of the PROCESS macro with 10,000 bootstrap samples. Mediation analysis diagrams are depicted in Fig. 2. The path estimates (direct, indirect, and total effects) of the proposed model along with 95% confidence intervals generated through the bootstrapping method are presented in Table 8.

**Table 8:** Path coefficients for mediation model

Effect	Paths	B	SE	t	p	LLCI	ULCI
*A: SAI, All Participants (N = 156, bootstrap = 10,000)*							
Total effect (c)	Resonance frequency power change → SAI change	0.0019	0.0148	0.1305	0.8964	− 0.0273	0.0311
Direct effect (c′)	Resonance frequency power change → SAI change	0.0089	0.0148	0.6018	0.5482	− 0.0203	0.0381
Indirect effect (ab)	Resonance frequency power change → Resting HRV Change → SAI change	− 0.007	0.0146			− **0.0528**	− **0.0013**
*B: SAI, Osc+ (N = 84, bootstrap = 10,000)*							
Total effect (c)	Resonance frequency power change → SAI Change	− 0.0466	0.0515	− 0.9057	0.3678	− 0.149	0.0558
Direct effect (c′)	Resonance frequency power change → SAI change	0.0124	0.0559	0.2222	0.8248	− 0.0988	0.1236
Indirect effect (ab)	Resonance frequency power change → Resting HRV Change → SAI change	− 0.059	0.0363			− **0.149**	− **0.0043**
*C: POMS, All Participants (N = 154, bootstrap = 10,000)*							
Total effect (c)	Resonance frequency power change → POMS change	0.0014	0.0096	0.1438	0.8859	− 0.0176	0.0204
Direct effect (c′)	Resonance frequency power change → POMS change	0.0058	0.0096	0.6004	0.5492	− 0.0133	0.0248
Indirect effect (ab)	Resonance frequency power change → Resting HRV Change → POMS change	− 0.0044	0.0116			− **0.0402**	− **0.0009**
*D: POMS, Osc+ (N = 82, bootstrap = 10,000)*							
Total effect (c)	Resonance frequency power change → POMS change	− 0.0061	0.0344	− 0.1768	0.8601	− 0.0745	0.0623
Direct effect (c′)	Resonance frequency power change → POMS change	0.0397	0.0369	1.0761	0.2852	− 0.0338	0.1132
Indirect effect (ab)	Resonance frequency power change → Resting HRV Change → POMS change	− 0.0458	0.0222			− **0.1004**	− **0.0133**

Values in bold indicate significant mediating effects

SE, standard error; LLCI, lower limit of the 95% confidence interval; ULCI, upper limit of the 95% confidence interval

First, we examined the mediation model for SAI for all participants. In this model with SAI as the dependent variable (Fig. 2A; Table 8A), the total effect was statistically insignificant, c = 0.0019, *p* = 0.896, 95% CI [− 0.0273, 0.0311]. The direct effect was also insignificant, c′ = 0.0089, *p* = 0.548, 95% CI [− 0.0203, 0.0381], but the indirect effect was significant, ab = − 0.007, 95% boot CI [− 0.0528, − 0.0013]. This pattern indicates a full mediation, in which increasing resonance frequency power during practice sessions can influence the SAI emotion score via changes in resting HRV, indicating a significant indirect effect (Baron & Kenny, 1986; Hayes, 2017). When we examined the mediation model for SAI for the Osc+ condition and Osc− condition separately (Fig. 2B for Osc+ , Fig. 2C for Osc−, and Table 8B), the total effect was not statistically significant for Osc+ condition, c = − 0.0466, *p* = 0.367, 95% CI [− 0.149, 0.0558]. The direct effect was also not significant, c′ = 0.0124, *p* = 0.3678, 95% CI [− 0.0988, 0.1236], but the indirect effect was significant for the Osc+ condition, ab = − 0.059, 95% boot CI [− 0.149, − 0.0043]. Thus, the results suggested that for the Osc+ condition, the relationship was full mediation where the resonance frequency power’s change affects the resting HRV change and thus the SAI change, indicating a significant indirect effect (Fig. 2B). On the contrary, for the Osc− condition there was no significant mediation effect (Fig. 2C). To test for differences across the two conditions in mediation effects, we ran a moderated mediation model on SAI change for all participants, with condition variable as a moderator. The results showed the moderated mediation effect was significant; moderated mediation = − 0.038, BootSE = 0.023, Boot 95% CI [− 0.0911, − 0.0011]. Thus, the condition significantly affected the relationship between resonance frequency power’s change, resting HRV changes, and SAI changes.

Next, we examined the mediation model for POMS for all participants. For this model with POMS as the dependent variable (Fig. 2D; Table 8C), the total effect was not statistically significant, c = 0.0014, *p* = 0.8859, 95% CI [− 0.0176, 0.0204]. The direct effect was not significant, c′ = 0.0096, *p* = 0.549, 95% CI [− 0.0133, 0.0248], but the indirect effect was significant, ab = − 0.0044, 95% CI [− 0.0402, − 0.0009]. Thus, for this model, the relationship was full mediation where the resonance frequency power’s change affects the resting HRV change and thus the POMS emotion score, indicating a significant indirect effect. When we examined the mediation model for POMS changes separately by condition, for the Osc+ condition (Fig. 2E; Table 8D), the total effect was not statistically significant, c = − 0.0061, *p* = 0.86, 95% CI [− 0.0745, 0.0623]. The direct effect was also not significant, c′ = 0.0397, *p* = 0.285, 95% CI [− 0.0338, 0.1132], but the indirect effect was significant, ab = − 0.0458, 95% CI [− 0.1004, − 0.0133]. Thus, the relationships indicated full mediation where the resonance frequency power’s change affects the resonance frequency power change and thus the POMS emotion score, indicating a significant indirect effect. For the Osc− condition, there was no significant mediation effect (Fig. 2F). To test for condition differences in mediation effects, we ran the moderated mediation model on POMS change for all participants, with condition variable as a moderator. The results showed the moderated mediation effect was significant; moderated mediation = − 0.024, BootSE = 0.014, Boot 95% CI [− 0.0570, − 0.0014]. Thus, changes in heart rate oscillation during practice sessions affected POMS via its effects on resting HRV more in the Osc+ than in the Osc− condition.

Next, we examined the mediation model for TAI for all participants. There was no significant total effect and direct effect, but there was significant indirect effect, ab = − 0.0043, 95% CI [− 0.0423, − 0.0003]. But there were no significant indirect effects for Osc+ and Osc− conditions and no moderated mediation by condition.

Finally, we examined the mediation model for CESD for all participants. There were no significant mediating effects in CESD for all participants and Osc+ condition and Osc− conditions.

In summary, among the measures of negative emotions, SAI and POMS showed a significant mediation effect of changes in resting HRV in the relationship between HRV biofeedback training and emotional changes, especially in the Osc+ experimental condition. However, in the case of TAI and CESD, TAI showed a significant mediating effect for the entire sample, but not in the Osc+ condition, while CESD did not show a significant mediating effect for either the entire sample or the Osc+ condition. This is likely because SAI and POMS measure the state of emotions, whereas TAI and CESD measure more general traits. In subsequent additional mediation effect analyses, only SAI and POMS, which showed significant mediation effects in the experimental condition, were applied.

### Moderated Mediation Models by Age Group and Sex

To test for age differences in the mediation effect, we performed moderated mediation analysis with SAI and POMS changes as dependent variables, resonance frequency power change as an independent variable, resting HRV change as a mediator, and age group as a moderator, controlling for sex. When we added age group as a moderator, we found no significant moderated mediation effects on SAI changes, effect = 0.0346, 95% boot CI [− 0.0156, 0.0764]. Conditional indirect effects were significant for both age groups; for younger adults, effect = − 0.0413, 95% boot CI [− 0.0905, − 0.0056], and for older adults, effect = − 0.0067, 95% boot CI [− 0.0548, − 0.0014] (Table 9A). We also found no significant moderated mediation effects on POMS change, effect = 0.0219, 95% boot CI [− 0.0110, 0.0538]. Conditional indirect effects were significant in both age groups; for younger adults, effect = − 0.0261, 95% boot CI [− 0.0667, − 0.0031], and for older adults, effect = − 0.0042, 95% boot CI [− 0.0406, − 0.0009] (Table 9D). Thus, using moderated mediation models, we did not find any age group differences in mediation effects on SAI and POMS changes.

**Table 9:** Path coefficients for moderated mediation model with age group as a moderator (N = 156, bootstrap = 10,000)

Effect	Paths	B	SE	t	p	LLCI	ULCI
*A: SAI, All Participants (N* = *156, bootstrap* = *10,000)*							
Direct effect	Resonance frequency power change → SAI Change	0.0089	0.0148	0.6018	0.5482	− 0.0203	0.0381
Indirect effect of Male	Resonance frequency power change → Log RMSSD change → SAI6 (Male)	− 0.0413	0.0218			− **0.0905**	− **0.0056**
Indirect effect of Female	Resonance frequency power change → Log RMSSD change → SAI6 (Female)	− 0.0067	0.0147			− **0.0548**	− **0.0014**
*B: SAI, Osc+ (N* = *84, bootstrap* = *10,000)*							
Direct effect	Resonance frequency power change → SAI Change	0.0124	0.0559	0.2222	0.8248	− 0.0988	0.1236
Indirect effect of Male	Resonance frequency power change → Log RMSSD change → SAI6 (Male)	− 0.0553	0.0383			− 0.1444	0.0062
Indirect effect of Female	Resonance frequency power change → Log RMSSD change → SAI6 (Female)	− 0.071	0.0433			− **0.1778**	− **0.0051**
*C: SAI, Osc− (N* = *72, bootstrap* = *10,000)*							
Direct effect	Resonance frequency power change → SAI Change	0.0124	0.0156	0.7907	0.4319	− 0.0188	0.0436
Indirect effect of Male	Resonance frequency power change → Log RMSSD change → SAI6 (Male)	− 0.0436	0.0425			− 0.1371	0.0287
Indirect effect of Female	Resonance frequency power change → Log RMSSD change → SAI6 (Female)	− 0.0018	0.0076			− 0.0241	0.0024
*D: POMS, All Participants (N* = *154, bootstrap* = *10,000)*							
Direct effect	Resonance frequency power change → SAI Change	0.0058	0.0096	0.6004	0.5492	− 0.0133	0.0248
Indirect effect of Male	Resonance frequency power change → Log RMSSD change → SAI6 (Male)	− 0.0261	0.0168			− **0.0667**	− **0.0031**
Indirect effect of Female	Resonance frequency power change → Log RMSSD change → SAI6 (Female)	− 0.0042	0.0113			− **0.0406**	− **0.0009**
*E: POMS, Osc+ (N* = *82, bootstrap* = *10,000)*							
Direct effect	Resonance frequency power change → SAI Change	0.0397	0.0369	1.0761	0.2852	− 0.0338	0.1132
Indirect effect of Male	Resonance frequency power change → Log RMSSD change → SAI6 (Male)	− 0.0427	0.0306			− **0.118**	− **0.0001**
Indirect effect of Female	Resonance frequency power change → Log RMSSD change → SAI6 (Female)	− 0.0547	0.0257			− **0.1157**	− **0.014**
*F: POMS, Osc− (N* = *72, bootstrap* = *10,000)*							
Direct effect	Resonance frequency power change → SAI Change	0.0039	0.0094	0.4185	0.677	− 0.0149	0.0227
Indirect effect of Male	Resonance frequency power change → Log RMSSD change → SAI6 (Male)	− 0.0148	0.0295			− 0.0878	0.0281
Indirect effect of Female	Resonance frequency power change → Log RMSSD change → SAI6 (Female)	− 0.006	0.0055			− 0.0158	0.0027

Values in bold indicate significant mediating effects

SE, standard error; LLCI, lower limit of the 95% confidence interval; ULCI, upper limit of the 95% confidence interval

When we applied a moderated mediation model separately by condition with age group as a moderator, we found no significant moderated mediation effect on SAI in Osc+ and Osc− conditions; effect = − 0.0158, 95% boot CI [− 0.1194, 0.0500] for Osc+ condition and effect = 0.0418, 95% boot CI [− 0.0273, 0.1293] for Osc− condition. For SAI in the Osc+ and Osc− condition, conditional indirect effects were reported in Table 9B, C. Similarly, we found no significant moderated mediation effect on POMS in Osc+ and Osc− conditions; effect = − 0.0120, 95% boot CI [− 0.0745, 0.0510] for Osc+ condition and effect = 0.0142, 95% boot CI [− 0.0265, 0.0806] for Osc− condition. For POMS in the Osc+ and Osc− condition, conditional indirect effects were reported in Table 9E, F. Thus, there were no significant age group differences in mediation models in Osc+ and Osc− conditions; Both age groups showed significant mediating effects in Osc+ condition and neither age group showed mediating effects in Osc− condition.

Similarly, we tested sex differences in mediation effects. To test for sex differences in the mediation effect, we performed moderated mediation analysis with SAI and POMS changes as dependent variables, resonance frequency power change as an independent variable, resting HRV change as a mediator, and sex as a moderator, controlling for age. When we added sex as a moderator, we found no significant moderated mediation effects on SAI changes, effect = − 0.0287, 95% boot CI [− 0.0634, 0.0318]. Conditional indirect effects were significant for both male and female groups; for the male group, effect = − 0.0025, 95% boot CI [− 0.0683, − 0.0005], and for the female group, effect = − 0.0312, 95% boot CI [− 0.0717, − 0.0058] (Table 10A). We also found no significant moderated mediation effects on POMS change, effect = − 0.0182, 95% boot CI [− 0.0392, 0.0269]. Conditional indirect effects were significant in both sex groups; for the male group, effect = − 0.0016, 95% boot CI [− 0.0568, − 0.0003], and for the female group, effect = − 0.0198, 95% boot CI [− 0.0490, − 0.0043] (Table 10D). Thus, using moderated mediation models, we did not find any sex differences in mediation effects on SAI and POMS changes.

**Table 10:** Path coefficients for moderated mediation model with sex as a moderator (N = 156, bootstrap = 10,000)

Effect	Paths	B	SE	t	p	LLCI	ULCI
*A: SAI, All Participants (N* = *156, bootstrap* = *10,000)*							
Direct effect	Resonance frequency power change → SAI Change	0.0086	0.0146	0.5858	0.5589	− 0.0203	0.0374
Indirect effect of Male	Resonance frequency power change → Log RMSSD change → SAI6 (Male)	− 0.0025	0.0187			− **0.0683**	− **0.0005**
Indirect effect of Female	Resonance frequency power change → Log RMSSD change → SAI6 (Female)	− 0.0312	0.017			− **0.0717**	− **0.0058**
*B: SAI, Osc+ (N* = *84, bootstrap* = *10,000)*							
Direct effect	Resonance frequency power change → SAI Change	0.0245	0.0536	0.4568	0.6491	− 0.0822	0.1311
Indirect effect of Male	Resonance frequency power change → Log RMSSD change → SAI6 (Male)	− 0.0826	0.0545			− **0.2151**	− **0.0021**
Indirect effect of Female	Resonance frequency power change → Log RMSSD change → SAI6 (Female)	− 0.0583	0.0389			− **0.1616**	− **0.0038**
*C: SAI, Osc− (N* = *72, bootstrap* = *10,000)*							
Direct effect	Resonance frequency power change → SAI Change	0.0099	0.0156	0.6354	0.5273	− 0.0212	0.0409
Indirect effect of Male	Resonance frequency power change → Log RMSSD change → SAI6 (Male)	− 0.0012	0.018			− 0.0574	0.0018
Indirect effect of Female	Resonance frequency power change → Log RMSSD change → SAI6 (Female)	− 0.014	0.0208			− 0.0726	0.0092
*D: POMS, All Participants (N* = *154, bootstrap* = *10,000)*							
Direct effect	Resonance frequency power change → SAI Change	0.0073	0.0096	0.7648	0.4456	− 0.0116	0.0262
Indirect effect of Male	Resonance frequency power change → Log RMSSD change → SAI6 (Male)	− 0.0016	0.0156			− **0.0568**	− **0.0003**
Indirect effect of Female	Resonance frequency power change → Log RMSSD change → SAI6 (Female)	− 0.0198	0.0114			− **0.049**	− **0.0043**
*E: POMS, Osc+ (N* = *82, bootstrap* = *10,000)*							
Direct effect	Resonance frequency power change → SAI Change	0.0666	0.0371	1.7956	0.0764	− 0.0072	0.1403
Indirect effect of Male	Resonance frequency power change → Log RMSSD change → SAI6 (Male)	− 0.068	0.0422			− **0.1719**	− **0.0097**
Indirect effect of Female	Resonance frequency power change → Log RMSSD change → SAI6 (Female)	− 0.048	0.0268			− **0.1187**	− **0.0119**
*F: POMS, Osc− (N* = *72, bootstrap* = *10,000)*							
Direct effect	Resonance frequency power change → SAI Change	0.0019	0.0095	0.1994	0.8425	− 0.017	0.0208
Indirect effect of Male	Resonance frequency power change → Log RMSSD change → SAI6 (Male)	− 0.0004	0.0132			− 0.0406	0.0022
Indirect effect of Female	Resonance frequency power change → Log RMSSD change → SAI6 (Female)	− 0.0048	0.0132			− 0.0403	0.0119

Values in bold indicate significant mediating effects

SE, standard error; LLCI, lower limit of the 95% confidence interval; ULCI, upper limit of the 95% confidence interval

When we applied moderated mediation model separately by condition with sex as a moderator, we found no significant moderated mediation effect on SAI in Osc+ and Osc− conditions; effect = 0.0243, 95% boot CI [− 0.0746, 0.1363] for Osc+ condition and effect = − 0.0128, 95% boot CI [− 0.0655, 0.344] for Osc− condition. For SAI in the Osc+ and Osc− conditions, conditional indirect effects were reported in Table 10B, C. Similarly, we found no significant moderated mediation effect on POMS in Osc+ and Osc− conditions; effect = 0.0200, 95% boot CI [− 0.0522, 0.1128] for Osc+ condition and effect = − 0.0044, 95% boot CI [− 0.0340, 0.0264] for Osc− condition. For POMS in the Osc+ and Osc− condition, conditional indirect effects were reported in Table 10E, F. Thus, there were no significant sex group differences in mediation models in Osc+ and Osc− conditions; both age groups showed significant mediating effects in Osc+ condition and neither sex group showed mediating effects in Osc− condition.

### Sequential Mediation Model

**Fig. 3:**
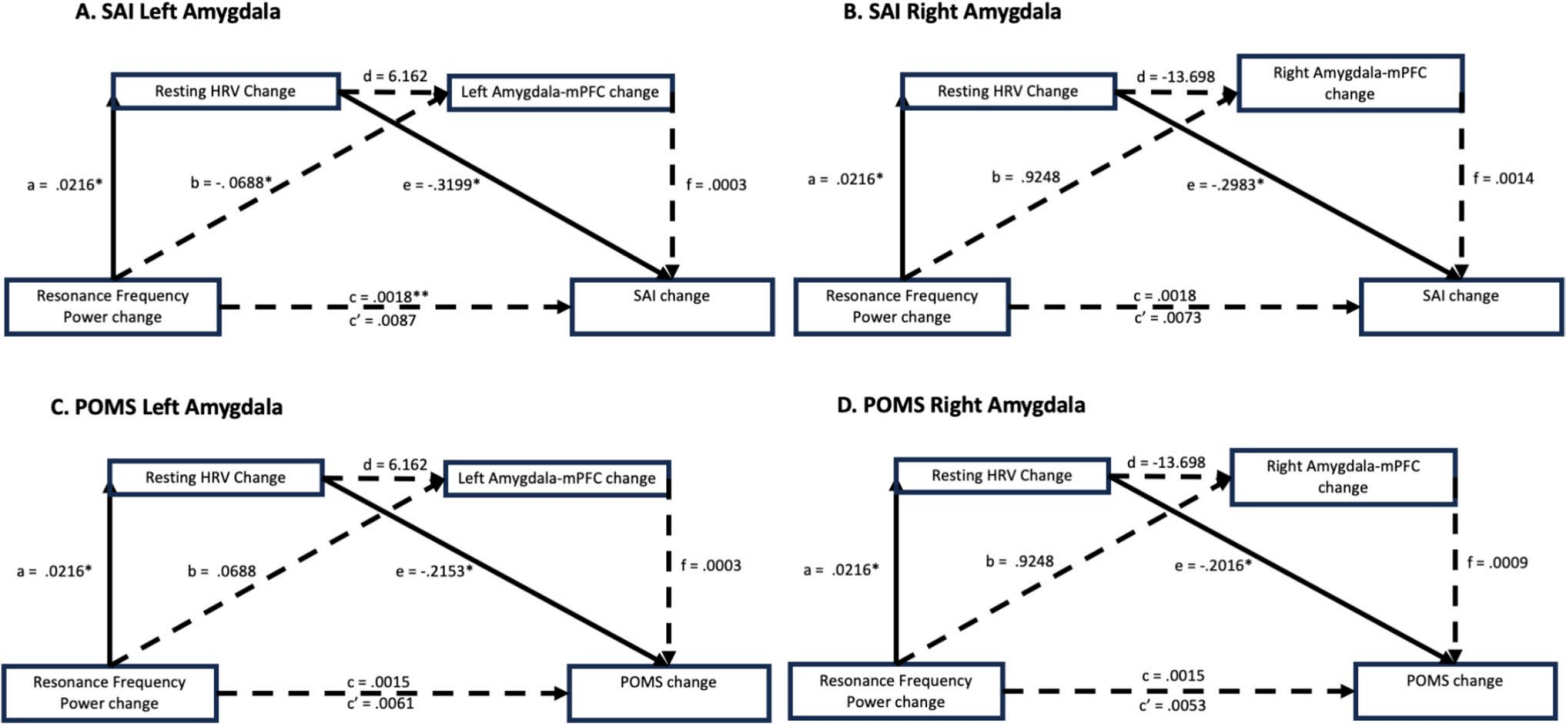
Sequential mediation models of SAI and POMS changes and Amygdala-mPFC connectivity change on the relationships between log RMSSD change and Resonance Frequency Power change. **a**–**c**, **c'**, **d**–**f** are expressed as the unstandardized regression coefficient. *p < 0.05; **p < 0.01

In the sequential mediation model for SAI and left amygdala-mPFC connectivity change, the total effect (c) of the resonance frequency power change on the SAI was not significant, B = 0.0018, 95% CI [− 0.0267, 0.0303]; Fig. 3 and Table 11. The direct effect (c') was also not significant, B = 0.0087, 95% CI [− 0.0198, 0.0372]. The total indirect effect was significant, B = − 0.0069, 95% CI [− 0.0562, − 0.0012]. The specific indirect effect through log RMSSD change (ae) was not significant, ae = 0.0069, 95% CI [− 0.0558, − 0.0013], and the indirect effect through left amygdala-mPFC connectivity change (bf) was not significant, bf = 0, 95% CI [− 0.0022, 0.0016]. The specific indirect effect through both log RMSSD change and left amygdala-mPFC connectivity change (adf) was also not significant, adf = 0, 95% CI [− 0.0003, 0.0013].

**Table 11:** Path coefficients for mediation model (N = 145, bootstrap = 10,000)

Effect	Paths	B	SE	t	p	LLCI	ULCI
A: SAI Left Amygdala							
Total effect (c)	Resonance frequency power change → SAI6 change	0.0018	0.0144	0.1239	0.9015	− 0.0267	0.0303
Direct effect (c′)	Resonance frequency power change → SAI6 change	0.0087	0.0144	0.6023	0.548	− 0.0198	0.0372
Indirect effects	Total indirect effect	− 0.0069	0.0155			− **0.0562**	− **0.0012**
(ae)	Resonance frequency power change → Log RMSSD change → SAI6 change	0.0069	0.0154			− **0.0558**	− **0.0013**
(bf)	Resonance frequency power change → Left amygdala-mPFC connectivity change → SAI6 change	0	0.0011			− 0.0022	0.0016
(adf)	Resonance frequency power change → Log RMSSD change → Left amygdala-mPFC connectivity change → SAI6 change	0	0.0004			− 0.0003	0.0013
*B: SAI Right Amygdala*							
Total effect (c)	Resonance frequency power change → SAI6 change	0.0018	0.0144	0.1239	0.9015	− 0.0267	0.0303
Direct effect (c′)	Resonance frequency power change → SAI6 change	0.0073	0.0144	0.5115	0.6098	− 0.021	0.0372
Indirect effects	Total indirect effect	− 0.0056	0.0136			− **0.049**	− **0.0002**
(ae)	Resonance frequency power change → Log RMSSD change → SAI6 change	0.0065	0.0144			− **0.0521**	− **0.0008**
(bf)	Resonance frequency power change → Right amygdala-mPFC connectivity change → SAI6 change	0.0013	0.0047			− 0.0028	0.0149
(adf)	Resonance frequency power change → Log RMSSD change → Right amygdala-mPFC connectivity change → SAI6 change	− 0.0004	0.0018			− 0.0062	0.0011
*C: POMS Left Amygdala*							
Total effect (c)	Resonance frequency power change → POMS6 change	0.0015	0.0097	0.1525	0.879	− 0.0176	0.0206
Direct effect (c′)	Resonance frequency power change → POMS6 change	0.0061	0.0097	0.6326	0.528	− 0.013	0.0252
Indirect effects	Total indirect effect	− 0.0046	0.0127			− **0.0445**	− **0.001**
(ae)	Resonance frequency power change → Log RMSSD change → SAI6 change	− 0.0047	0.0125			− **0.0552**	− **0.0011**
(bf)	Resonance frequency power change → Left amygdala-mPFC connectivity change → POMS6 change	0	0.0008			− 0.002	0.0006
(adf)	Resonance frequency power change → Log RMSSD change → Left amygdala-mPFC connectivity change → POMS6 change	0	0.0003			− 0.0001	0.001
*D: POMS Right Amygdala*							
Total effect (c)	Resonance frequency power change → POMS6 change	0.0015	0.0097	0.1525	0.879	− 0.0176	0.0206
Direct effect (c′)	Resonance frequency power change → POMS6 change	0.0053	0.0096	0.5481	0.5845	− 0.0138	0.0243
Indirect effects	Total indirect effect	− 0.0038	0.0115			− **0.0407**	− **0.0004**
(ae)	Resonance frequency power change → Log RMSSD change → SAI6 change	− 0.0044	0.012			− **0.043**	− **0.0009**
(bf)	Resonance frequency power change → Right amygdala-mPFC connectivity change → POMS6 change	0.0008	0.0024			− 0.0008	0.0077
(adf)	Resonance frequency power change → Log RMSSD change → Right amygdala-mPFC connectivity change → POMS6 change	− 0.0003	0.0008			− 0.0026	0.0008

Values in bold indicate significant mediating effects

SE, standard error; LLCI, lower limit of the 95% confidence interval; ULCI, upper limit of the 95% confidence interval

In the second sequential mediation model for SAI and right amygdala-mPFC connectivity change, neither the total effect (c) of the resonance frequency power change on the SAI, B = 0.0018, 95% CI [− 0.0267, 0.0303], nor the direct effect (c'), B = 0.0073, 95% CI [− 0.0210, 0.0357], was significant. However, the total indirect effect was significant, B = − 0.0056, 95% CI [− 0.0490, − 0.0002]. No specific indirect effects through log RMSSD change (ae), ae = 0.0065, 95% CI [− 0.0521, − 0.0008] or right amygdala-mPFC connectivity change (bf), bf = 0.0013, 95% CI [− 0.0028, 0.0149] were significant. The sequential indirect path through log RMSSD change and right amygdala-mPFC connectivity change (adf) was not significant, adf = − 0.0004, 95% CI [− 0.0062, 0.0011].

The analysis for POMS and left amygdala-mPFC connectivity change indicated that the total effect (c) of resonance frequency power change on POMS was not significant, B = 0.0015, 95% CI [− 0.0176, 0.0206]. The direct effect (c') was also non-significant, B = 0.0061, 95% CI [− 0.0130, 0.0252]. The total indirect effect was significant, B = − 0.0046, 95% CI [− 0.0445, − 0.0010]. The specific indirect effects through log RMSSD change (ae), ae = − 0.0047, 95% CI [− 0.0552, − 0.0011] and left amygdala-mPFC connectivity change (bf path), bf = 0, 95% CI [− 0.002, 0.0006] were not significant. Furthermore, the adf path was also not significant, adf = 0, 95% CI [− 0.0001, 0.001].

For the sequential model for POMS and right amygdala-mPFC connectivity change, the analysis indicated that the total effect (c) of resonance frequency power change on POMS was not significant, B = 0.0015, 95% CI [− 0.0176, 0.0206]. The direct effect (c’) of resonance frequency power change on POMS was also non-significant, B = 0.0053, 95% CI [− 0.0138, 0.0243]. The total indirect effect through log RMSSD change and right amygdala-mPFC connectivity change was significant, B = − 0.0038, CI [− 0.0407, − 0.0004]. No specific indirect effects for ae, bf, and adf paths were reported. The specific indirect effects through log RMSSD change (ae), ae = − 0.0044, 95% CI [− 0.043, − 0.0009] was significant. However, the left amygdala-mPFC connectivity change (bf path), bf = 0.0008, 95% CI [− 0.0008, 0.0077] were not significant. Furthermore, the adf path was also not significant, adf = − 0.0003, 95% CI [− 0.0026, 0.0008].

## Discussion

We initially examined the associations between resting HRV, two emotional trait scores (CESD and TAI) and two emotional state scores (SAI and POMS) at pre-intervention. Results indicated that log RMSSD showed significant negative correlations with POMS scores at pre-intervention. When we explored potential age-related differences in HRV and emotion scores by splitting the data by age groups, younger adults showed no significant correlations between HRV and emotion scores, while older adults displayed significant negative correlations between SAI and POMS and HRV indexed by log RMSSD, log HF-power, and log LF-power.

We next found that resting HRV change and negative emotion changes from pre-intervention to post-intervention were correlated. For all participants, there was a significant negative partial correlation between log RMSSD change and the negative emotion score changes. In the Osc+ condition, significant negative correlations were observed for SAI and POMS. In the Osc− condition, there were no significant correlations. Thus, in the context of an HRV biofeedback intervention to increase heart rate oscillations in daily practice sessions, post-intervention increased log RMSSD during non-practice resting is associated with post-intervention decreased anxiety (SAI) and decreased negative mood (POMS).

We then conducted mediation analyses whether the relationship between training performance and negative emotions was mediated by change of resting HRV. Two separate mediation models for SAI and POMS were examined for all participants combined, and separately for the Osc+ and Osc− conditions. Results indicated that the effects of resonance frequency power during practice sessions on negative emotion changes were mediated by resting HRV changes from pre-to-post intervention. This mediation effect was moderated by condition, such that only the Osc+ condition showed significant mediation effects for both emotion scores. Lastly, we extended the mediation models by adding left or right amygdala-mPFC connectivity change as a second mediator. The results showed that there was no significant sequential mediation effect of amygdala-mPFC connectivity on SAI or POMS; there was only a significant mediation effect of resting HRV change on the negative emotion changes induced by HRV biofeedback training.

Based on the analysis divided by age and sex groups, the number of significant correlations in the baseline correlation analysis was higher in the older than younger adults and in the female than the male group. However, the results of testing the difference in correlation coefficients did not show significant differences between age or sex groups. The moderated mediation analysis revealed that there were no significant moderated mediation effects by age group or gender. This indicates that the effect of HRV biofeedback on emotions via vmHRV did not differ by age or gender groups, showing the same mediating effect across these groups.

In summary, our study provides valuable insights into the associations between resting RMSSD, emotion scores, and the impact of HRV biofeedback training. In the HRV-ER clinical trial, no significant condition differences were found in changes in self-rated emotions (Nashiro et al., 2023). However, this study showed that improvements in daily emotional states were mediated by intervention-induced increases in vagal HRV during resting states. The findings highlight the mediating role of resting HRV in the relationship between HRV biofeedback training and negative emotions, and these results were consistent across both younger and older groups, as well as among both females and males. These results shed light on the potential mechanisms underlying the effectiveness of HRV biofeedback training in improving emotional well-being, particularly in older adults, and emphasize the importance of considering intervention-specific effects when analyzing mediation pathways.

## Supplementary Information

Below is the link to the electronic supplementary material.

Supplementary file1 (DOCX 568 KB)

## Data Availability

The empirical data used for this paper is available in the public repository, OpenNeuro, “HRV-ER” (https://openneuro.org/datasets/ds003823) before publication. The codes for other statistical analyses are available upon request from the corresponding author.
