# Supplementary material for: Changes in Negative Emotions Across Five Weeks of HRV Biofeedback Intervention were Mediated by Changes in Resting Heart Rate Variability
Source: Appl Psychophysiol Biofeedback. 2024 Nov 8;50(1):25–48. doi: 10.1007/s10484-024-09674-x (PMC11882736; doi:10.1007/s10484-024-09674-x)
Supplement: Supplementary file 1 — Supplementary file1 (DOCX 568 KB) [file 10484_2024_9674_MOESM1_ESM.docx]

**Supplementary Figure 1**

*Numbers of participants in each intervention condition, how many participants completed each measure, and how many were included vs. excluded in each analysis.*


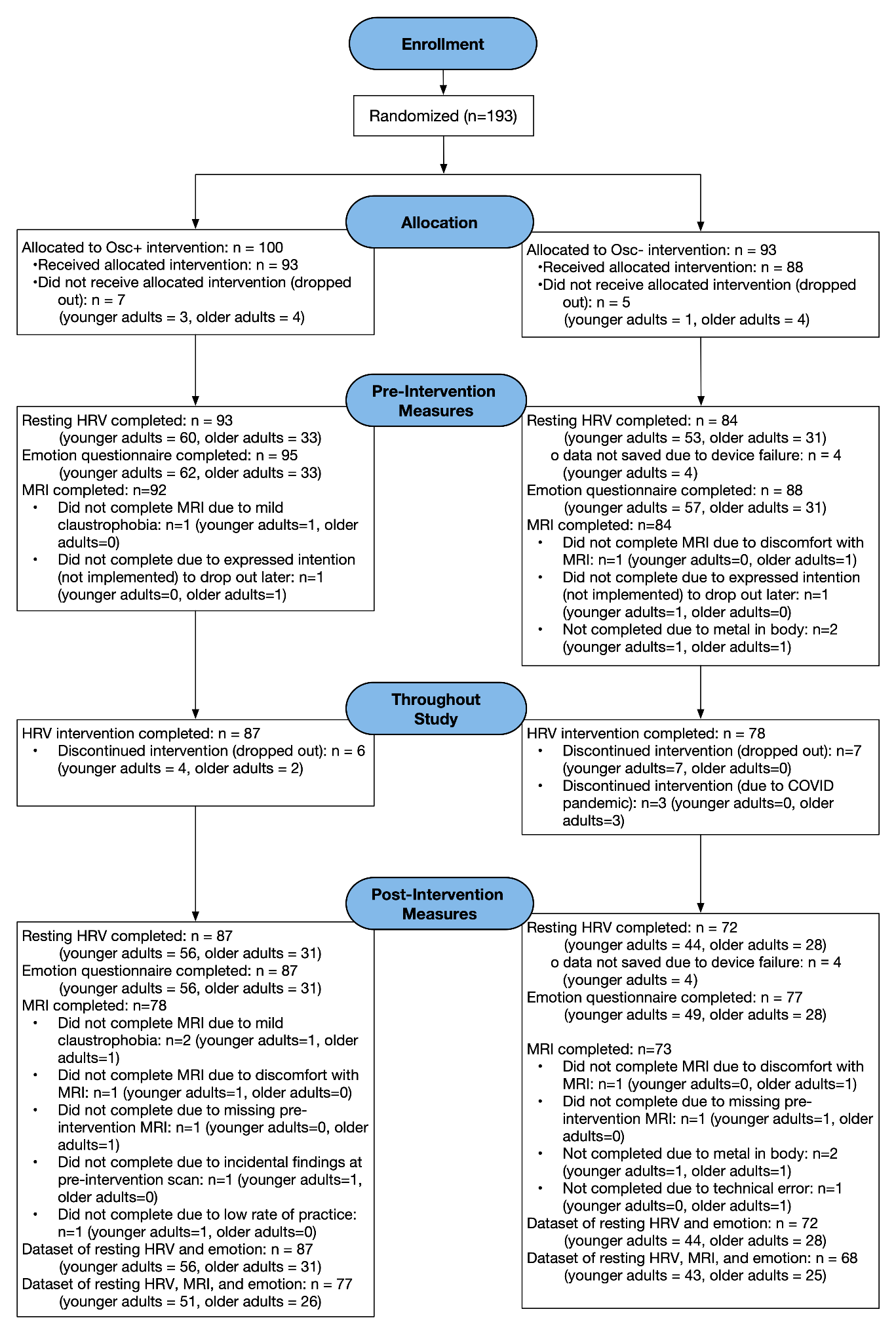


**Supplementary Table 1**

| *Male YA Correlations* | | | | | | | | | |
| --- | --- | --- | --- | --- | --- | --- | --- | --- | --- |
| Correlations |  | Mean HR at pre | Log RMSSD at pre | Log HF-power at pre | Log LF-power at pre | CESD at pre | TAI at pre | SAI at pre | POMS at pre |
| Mean HR at pre | *r* | 1 |  |  |  |  |  |  |  |
|  | *p* |  |  |  |  |  |  |  |  |
|  | *df* | 0 |  |  |  |  |  |  |  |
| Log RMSSD at pre | *r* | -0.47*** | 1 |  |  |  |  |  |  |
|  | *p* | <.001 |  |  |  |  |  |  |  |
|  | *df* | 47 | 0 |  |  |  |  |  |  |
| Log HF-power at pre | *r* | -0.50*** | 0.95*** | 1 |  |  |  |  |  |
|  | *p* | <.001 | <.001 |  |  |  |  |  |  |
|  | *df* | 47 | 47 | 0 |  |  |  |  |  |
| Log LF-power at pre | *r* | -0.36*** | 0.66*** | 0.61*** | 1 |  |  |  |  |
|  | *p* | 0.011 | <.001 | <.001 |  |  |  |  |  |
|  | *df* | 47 | 47 | 47 | 0 |  |  |  |  |
| CESD at pre | *r* | -0.02 | 0.01 | 0.07 | -0.14 | 1 |  |  |  |
|  | *p* | 0.890 | 0.959 | 0.614 | 0.357 |  |  |  |  |
|  | *df* | 47 | 47 | 47 | 47 | 0 |  |  |  |
| TAI at pre | *r* | -0.16 | 0.06 | 0.09 | -0.11 | 0.83*** | 1 |  |  |
|  | *p* | 0.273 | 0.674 | 0.520 | 0.434 | <.001 |  |  |  |
|  | *df* | 47 | 47 | 47 | 47 | 47 | 0 |  |  |
| SAI at pre | *r* | -0.11 | -0.02 | -0.03 | -0.06 | 0.64*** | 0.79*** | 1 |  |
|  | *p* | 0.473 | 0.899 | 0.825 | 0.682 | <.001 | <.001 |  |  |
|  | *df* | 47 | 47 | 47 | 47 | 47 | 47 | 0 |  |
| POMS at pre | *r* | -0.06 | -0.07 | -0.06 | -0.09 | 0.68*** | 0.74*** | 0.9*** | 1 |
|  | *p* | 0.699 | 0.626 | 0.674 | 0.546 | <.001 | <.001 | <.001 |  |
|  | *df* | 47 | 47 | 47 | 47 | 47 | 47 | 47 | 0 |

**p* < .05; ***p* < .01, ****p* < .001, 2-tailed.

**Supplementary Table 2**

| *Male OA Correlations* | | | | | | | | | | |
| --- | --- | --- | --- | --- | --- | --- | --- | --- | --- | --- |
| Correlations |  | Mean HR at pre | Log RMSSD at pre | Log HF-power at pre | Log LF-power at pre | CESD at pre | TAI at pre | SAI at pre | POMS at pre |  |
| Mean HR at pre | *r* | 1 |  |  |  |  |  |  |  |  |
|  | *p* |  |  |  |  |  |  |  |  |  |
|  | *df* | 0 |  |  |  |  |  |  |  |  |
| Log RMSSD at pre | *r* | -0.39 | 1 |  |  |  |  |  |  |  |
|  | *p* | 0.151 |  |  |  |  |  |  |  |  |
|  | *df* | 13 | 0 |  |  |  |  |  |  |  |
| Log HF-power at pre | *r* | -0.51 | 0.94*** | 1 |  |  |  |  |  |  |
|  | *p* | 0.054 | <.001 |  |  |  |  |  |  |  |
|  | *df* | 13 | 13 | 0 |  |  |  |  |  |  |
| Log LF-power at pre | *r* | -0.61* | 0.70** | 0.82*** | 1 |  |  |  |  |  |
|  | *p* | 0.016 | 0.003 | <.001 |  |  |  |  |  |  |
|  | *df* | 13 | 13 | 13 | 0 |  |  |  |  |  |
| CESD at pre | *r* | 0.25 | -0.44 | -0.25 | -0.25 | 1 |  |  |  |  |
|  | *p* | 0.364 | 0.103 | 0.369 | 0.366 |  |  |  |  |  |
|  | *df* | 13 | 13 | 13 | 13 | 0 |  |  |  |  |
| TAI at pre | *r* | 0.28 | -0.37 | -0.19 | -0.2 | 0.95*** | 1 |  |  |  |
|  | *p* | 0.304 | 0.175 | 0.495 | 0.485 | <.001 |  |  |  |  |
|  | *df* | 13 | 13 | 13 | 13 | 13 | 0 |  |  |  |
| SAI at pre | *r* | 0.41 | -0.25 | -0.15 | -0.15 | 0.87*** | 0.9*** | 1 |  |  |
|  | *p* | 0.127 | 0.376 | 0.596 | 0.602 | <.001 | <.001 |  |  |  |
|  | *df* | 13 | 13 | 13 | 13 | 13 | 13 | 0 |  |  |
| POMS at pre | *r* | 0.35 | -0.36 | -0.23 | -0.14 | 0.90*** | 0.90*** | 0.88*** | 1 |  |
|  | *p* | 0.208 | 0.192 | 0.411 | 0.632 | <.001 | <.001 | <.001 |  |  |
|  | *df* | 13 | 13 | 13 | 13 | 13 | 13 | 13 | 0 |  |

**p* < .05; ***p* < .01, ****p* < .001, 2-tailed.

**Supplementary Table 3**

| *Female YA Correlations* | | | | | | | | | |
| --- | --- | --- | --- | --- | --- | --- | --- | --- | --- |
| Correlations |  | Mean HR at pre | Log RMSSD at pre | Log HF-power at pre | Log LF-power at pre | CESD at pre | TAI at pre | SAI at pre | POMS at pre |
| Mean HR at pre | *r* | 1 |  |  |  |  |  |  |  |
|  | *p* |  |  |  |  |  |  |  |  |
|  | *df* | 0 |  |  |  |  |  |  |  |
| Log RMSSD at pre | *r* | -0.46*** | 1 |  |  |  |  |  |  |
|  | *p* | 0.001 |  |  |  |  |  |  |  |
|  | *df* | 45 | 0 |  |  |  |  |  |  |
| Log HF-power at pre | *r* | -0.45** | 0.94*** | 1 |  |  |  |  |  |
|  | *p* | 0.002 | <.001 |  |  |  |  |  |  |
|  | *df* | 45 | 45 | 0 |  |  |  |  |  |
| Log LF-power at pre | *r* | -0.29* | 0.72*** | 0.71*** | 1 |  |  |  |  |
|  | *p* | 0.047 | <.001 | <.001 |  |  |  |  |  |
|  | *df* | 45 | 45 | 45 | 0 |  |  |  |  |
| CESD at pre | *r* | 0.36* | 0.01 | 0.04 | -0.01 | 1 |  |  |  |
|  | *p* | 0.012 | 0.956 | 0.810 | 0.960 |  |  |  |  |
|  | *df* | 45 | 45 | 45 | 45 | 0 |  |  |  |
| TAI at pre | *r* | 0.43** | -0.12 | -0.14 | -0.18 | 0.79*** | 1 |  |  |
|  | *p* | 0.003 | 0.409 | 0.353 | 0.238 | <.001 |  |  |  |
|  | *df* | 45 | 45 | 45 | 45 | 45 | 0 |  |  |
| SAI at pre | *r* | 0.23 | -0.06 | -0.08 | -0.08 | 0.65*** | 0.76*** | 1 |  |
|  | *p* | 0.128 | 0.686 | 0.602 | 0.594 | <.001 | <.001 |  |  |
|  | *df* | 45 | 45 | 45 | 45 | 45 | 45 | 0 |  |
| POMS at pre | *r* | 0.18 | -0.12 | -0.10 | -0.11 | 0.65*** | 0.61*** | 0.78*** | 1 |
|  | *p* | 0.218 | 0.409 | 0.525 | 0.446 | <.001 | <.001 | <.001 |  |
|  | *df* | 45 | 45 | 45 | 45 | 45 | 45 | 45 | 0 |

**p* < .05; ***p* < .01, ****p* < .001, 2-tailed.

**Supplementary Table 4**

| *Female OA Correlations* | | | | | | | | | |
| --- | --- | --- | --- | --- | --- | --- | --- | --- | --- |
| Correlations |  | Mean HR at pre | Log RMSSD at pre | Log HF-power at pre | Log LF-power at pre | CESD at pre | TAI at pre | SAI at pre | POMS at pre |
| Mean HR at pre | *r* | 1 |  |  |  |  |  |  |  |
|  | *p* |  |  |  |  |  |  |  |  |
|  | *df* | 0 |  |  |  |  |  |  |  |
| Log RMSSD at pre | *r* | -0.42** | 1 |  |  |  |  |  |  |
|  | *p* | 0.007 |  |  |  |  |  |  |  |
|  | *df* | 39 | 0 |  |  |  |  |  |  |
| Log HF-power at pre | *r* | -0.54*** | 0.96*** | 1 |  |  |  |  |  |
|  | *p* | <.001 | <.001 |  |  |  |  |  |  |
|  | *df* | 39 | 39 | 0 |  |  |  |  |  |
| Log LF-power at pre | *r* | -0.51*** | 0.81*** | 0.82*** | 1 |  |  |  |  |
|  | *p* | 0.001 | <.001 | <.001 |  |  |  |  |  |
|  | *df* | 39 | 39 | 39 | 0 |  |  |  |  |
| CESD at pre | *r* | 0.05 | -0.11 | -0.10 | -0.17 | 1 |  |  |  |
|  | *p* | 0.775 | 0.484 | 0.526 | 0.292 |  |  |  |  |
|  | *df* | 39 | 39 | 39 | 39 | 0 |  |  |  |
| TAI at pre | *r* | 0.04 | -0.19 | -0.19 | -0.13 | 0.72*** | 1 |  |  |
|  | *p* | 0.823 | 0.233 | 0.225 | 0.419 | 0.000 |  |  |  |
|  | *df* | 39 | 39 | 39 | 39 | 39 | 0 |  |  |
| SAI at pre | *r* | 0.32* | -0.32* | -0.35* | -0.39*** | 0.60*** | 0.61*** | 1 |  |
|  | *p* | 0.045 | 0.042 | 0.027 | 0.012 | <.001 | <.001 |  |  |
|  | *df* | 39 | 39 | 39 | 39 | 39 | 39 | 0 |  |
| POMS at pre | *r* | 0.35* | -0.34* | -0.37* | -0.35* | 0.55*** | 0.64*** | 0.83*** | 1 |
|  | *p* | 0.025 | 0.030 | 0.017 | 0.024 | <.001 | <.001 | <.001 |  |
|  | *df* | 39 | 39 | 39 | 39 | 39 | 39 | 39 | 0 |

**p* < .05; ***p* < .01, ****p* < .001, 2-tailed.
